# Maternal diet during early gestation influences postnatal taste activity–dependent pruning by microglia

**DOI:** 10.1084/jem.20212476

**Published:** 2023-09-21

**Authors:** Chengsan Sun, Shuqiu Zheng, Justin S.A. Perry, Geoffrey T. Norris, Mei Cheng, Fanzhen Kong, Rolf Skyberg, Jianhua Cang, Alev Erisir, Jonathan Kipnis, David L. Hill

**Affiliations:** 1 Department of Psychology, University of Virginia, Charlottesville, VA, USA; 2 Division of Nephrology, University School of Medicine, Charlottesville, VA, USA; 3 Immunology Program, Sloan Kettering Institute, Memorial Sloan Kettering Cancer Center, New York, NY, USA; 4 Department of Immunology, University of Washington, Seattle, WA, USA; 5 Department of Health and Disease Management, Binzhou Medical University, Yantai, China; 6 Department of Anatomy, Binzhou Medical University, Yantai, China; 7 Institute of Neuroscience, University of Oregon, Eugene, OR, USA; 8 Departments of Psychology and Biology, University of Virginia, Charlottesville, VA, USA; 9 Department of Pathology and Immunology, Washington University, St. Louis, MO, USA

## Abstract

A key process in central sensory circuit development involves activity-dependent pruning of exuberant terminals. Here, we studied gustatory terminal field maturation in the postnatal mouse nucleus of the solitary tract (NST) during normal development and in mice where their mothers were fed a low NaCl diet for a limited period soon after conception. Pruning of terminal fields of gustatory nerves in controls involved the complement system and is likely driven by NaCl-elicited taste activity. In contrast, offspring of mothers with an early dietary manipulation failed to prune gustatory terminal fields even though peripheral taste activity developed normally. The ability to prune in these mice was rescued by activating myeloid cells postnatally, and conversely, pruning was arrested in controls with the loss of myeloid cell function. The altered pruning and myeloid cell function appear to be programmed before the peripheral gustatory system is assembled and corresponds to the embryonic period when microglia progenitors derived from the yolk sac migrate to and colonize the brain.

## Teaser

Microglia aid in refining central gustatory circuits during normal postnatal development. This process is arrested by merely restricting the mother’s dietary sodium during a limited, early prenatal period when microglia progenitors migrate from the yolk sac to the brain.

## Introduction

A hallmark of developing sensory systems is that an excess of afferent terminals and synapses are formed in the central nervous system (CNS) during early development, and then over time, weak or inappropriate synapses are pruned by activity-dependent molecular mechanisms (Hooks and Chen, 2006; Leake et al., 2002; Sakai, 2020; Sretavan et al., 1988). A key contributor to the pruning of these synapses is microglia, which have been elegantly shown to operate in the postnatal development of circuits carrying binocular visual information from the retina to the dorsal lateral geniculate nucleus (dLGN) in the thalamus (Schafer et al., 2012; Shatz, 2009; Stephan et al., 2012; Stevens et al., 2007). Moreover, the early prenatal formation of central neuronal circuits is organized by microglia (Ginhoux and Prinz, 2015; Pont-Lezica et al., 2014; Squarzoni et al., 2014). These discoveries highlight the involvement of microglia in both the prenatal establishment and the postnatal refinement of neuronal pathways throughout pre- and postnatal development.

**Figure 1.**
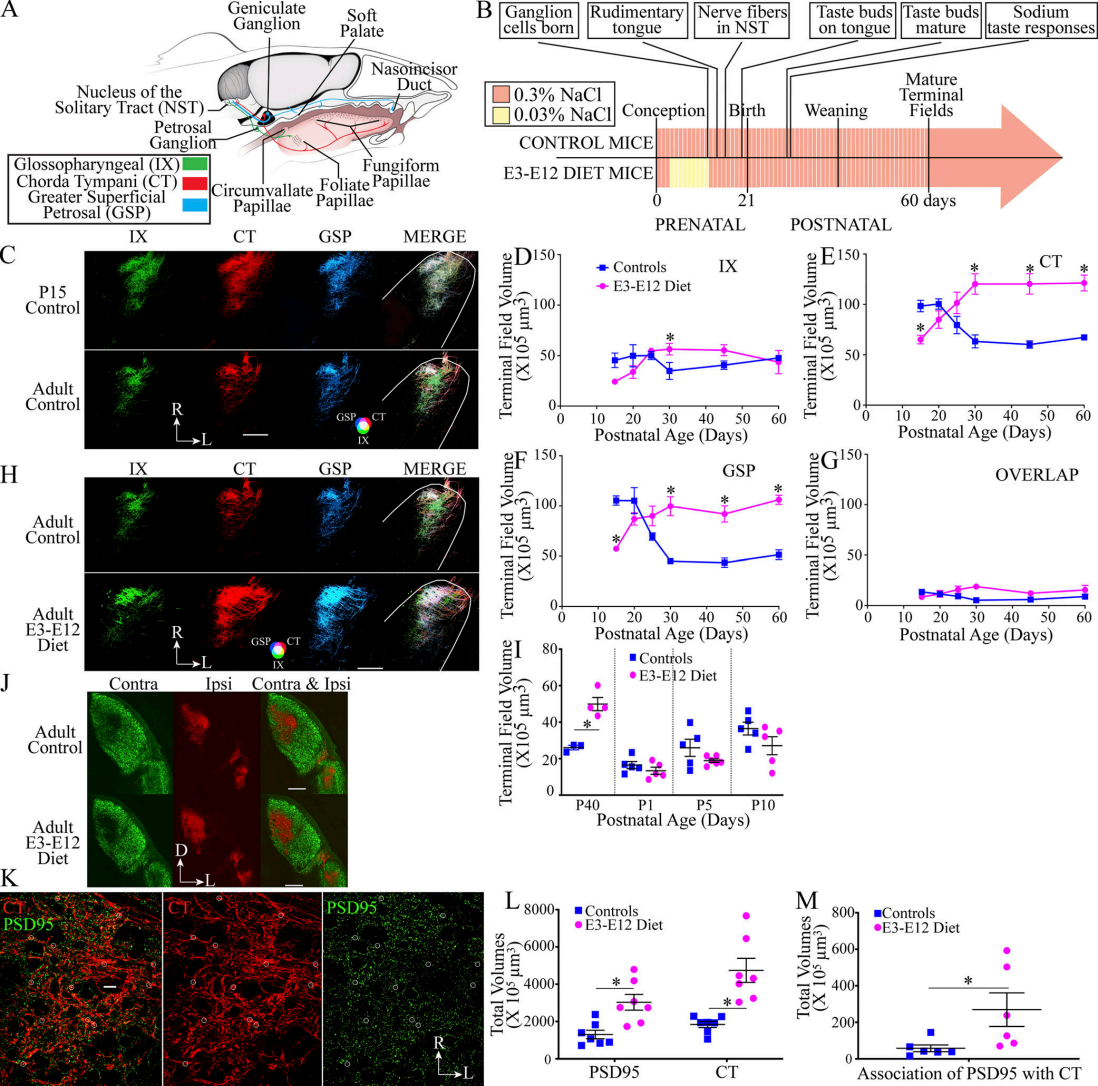
**Terminal field volumes selectively decrease with age in control mice and increase in E3–E12 diet mice.** Expanded terminal field sizes in the E3–E12 diet are associated with increased numbers of putative synapses. **(A)** Schematic of the peripheral gustatory system in a mouse showing the three nerves that carry taste information from taste buds on the tongue to the rostral portion of the NST. Cell somas of the three nerves are in the geniculate (CT and GSP) and petrosal (glossopharyngeal, IX) ganglia. The CT, GSP, and IX innervate taste buds located on the anterior tongue, palate, and posterior tongue, respectively. **(B)** Timeline of early embryonic dietary exposure period (sodium dietary restriction period denoted in yellow) and hallmarks of peripheral gustatory development. **(C and H)** Horizontal sections of labeled terminal fields of the IX (green), CT (red), GSP (blue), and the merged image (MERGE) in representative sections from the dorsal zone in P15 and adult control mice (C) and in adult control and adult E3–E12 diet mice (H). Refer to the color guide in the lower portion of C and H for overlap colors shown in the merged images. R, rostral; L, lateral. Scale bars in C and H = 200 µm. The photomicrographs used in C are duplicated for the dorsal zone in Fig. S1 A and the photomicrographs used in H are duplicated for the dorsal zone in Fig. S1 B. **(D–G)** Terminal field volumes of controls and E3–E12 diet mice for the IX (D), CT (E), and GSP (F) nerves and the overlapping fields of all three nerves (G). The numbers of mice/age for control and E3–E12 groups are, respectively: P15 = 5, 6; P20 = 6, 6; P25 = 5, 5; P30 = 5, 5; P45 = 4, 5; adult = 5, 5. **(I)** Terminal field volumes of controls and E3–E12 diet mice obtained through measurements of P2X2^+^ labeling in P40, P1, P5, and P10 mice. The numbers of mice/age for control and E3–E12 groups are, respectively: P40 = 3, 4; P1 = 5, 5; P5 = 5, 6; P10 = 5, 5. **(J)** Coronal sections of the right dLGN in adult control (*n* = 3) and E3–E12 diet mice (*n* = 3) showing the terminal field label for retinogeniculate projections from the contralateral (green) and ipsilateral (red) eye. Scale bar = 500 µm. D, dorsal; L, lateral. **(K)** Photomicrograph of an optical, horizontal section through the rostral NST. CT terminal field is shown in red, and PSD95^+^ labeling is shown in green. Examples of putative CT synapses are outlined by white circles. Scale bar (white) in center of left panel = 20 µm. R, rostral; L, lateral. **(L)** Volume of PSD^+^ and CT terminal field labeling within the region of interest in the NST of adult controls (PSD95, *n* = 7; CT, *n* = 6) and E3–E12 mice (PSD95, *n* = 7; CT, *n* = 6). **(M)** Volume of labels where PSD^+^ labeling was closely associated with CT terminals within the region of interest in the NST of controls (PSD95, *n* = 7; CT, *n* = 6) and E3–E12 mice (PSD95, *n* = 7; CT, *n* = 6). Data in D–G and I are shown as mean ± SEM. All statistical comparisons were multiple, unpaired *t* tests using the Holm–Sidak method for multiple comparisons. * denotes P < 0.05. Terminal field volumes (D–G)—IX: P30 control versus E3–E12, P = 0.02; CT: P values at P15, P30, P50, and P60 are 0.002, 0.0000043, 0.0000046, and 0.000001, respectively; GSP: P values at P15, P30, P50, and P60 are 0.000008, 0.0000018, 0.00004, and 0.0000017, respectively. **(I)** P = 0.003 for comparison at P40. **(L)** P = 0.02 for both comparisons. **(M)** P = 0.04. One observation/animal was made for all measures of terminal field volumes and immunohistochemistry.

Here, we used the developing gustatory system to expand and characterize neural–immune interactions driving normal sensory system circuit refinement, and importantly, begin to define events and factors in early embryonic development that direct the trajectory of these neural-immune processes. The developing rodent gustatory system is an especially useful model for examining these questions for multiple reasons: there is a large-scale and extended period of postnatal reorganization of multiple cranial nerve circuits carrying taste information from the tongue to the nucleus of the solitary tract (NST) in the brainstem (Fig. 1 A); the period of circuit reorganization coincides with an increase in peripheral functional taste sensitivity to sodium salts indicating an activity-dependent process(es) (Fig. 1 B; Ferrell et al., 1981; Hill and Almli, 1980; Hill et al., 1982; Mangold and Hill, 2008; Yamada, 1980); and these circuits show life-long functional and structural plasticity (Chang and Scott, 1984; Corson and Hill, 2011; Giza and Scott, 1983; Skyberg et al., 2017; Sun et al., 2018). The developmental process of postnatal circuit refinement can be short-circuited in offspring of mothers fed a sodium-deficient diet during a relatively brief period after conception (Krimm and Hill, 1997; Mangold and Hill, 2008). Unexpectedly, this prenatal sensitive period coincides when microglia progenitors derived from the yolk sac migrate to and colonize the brain (Ginhoux et al., 2010; Ginhoux and Prinz, 2015), but is over before the emergence of the tongue and geniculate ganglion neurons (Altman and Bayer, 1982; Paulson et al., 1985; cell somas of two key gustatory nerves; Fig. 1 B). Here, we will leverage this finding to link how environmental factors during very early embryonic development impact the neural/immune interactions driving postnatal central circuit refinement.

We find that gustatory terminal fields in control mice selectively reorganize between P20 and P30 in what appears to be an activity-dependent process and likely involves the classical complement cascade components of C1q and C3 and IL-34 and IL-33. We also show that myeloid function and failure to prune gustatory terminal fields are compromised in offspring of dams fed a low-sodium diet from 3 to 12 d after conception (i.e., during embryonic days 3–12 [E3–E12] for their offspring). These findings add to the relatively few studies showing that the immune system is involved in the normal developmental process of sensory circuit refinement and that environmental factors early in embryonic development are critical to these neuro-immune–driven processes throughout the animal’s life.

## Results

Earlier work in rats established the foundation for these studies, which is that offspring of mothers fed a sodium-restricted diet from 3 to 12 d after conception failed to prune gustatory terminal fields in the NST postnatally (Mangold and Hill, 2008). However, these earlier findings are limited because of the lack of molecular/cellular tools available for mechanistic studies with this species. Here, we first established the timing, magnitude, and identity of pruning of gustatory afferents in mice, thereby allowing the use of essential experimental tools and complementary findings in other systems. These results were key to appropriately designing experiments aimed at discovering mechanisms driving central circuit development.

### Total terminal field volumes in the NST selectively decrease with age in control mice and increase with age in E3–E12 diet mice

**Figure S1.**
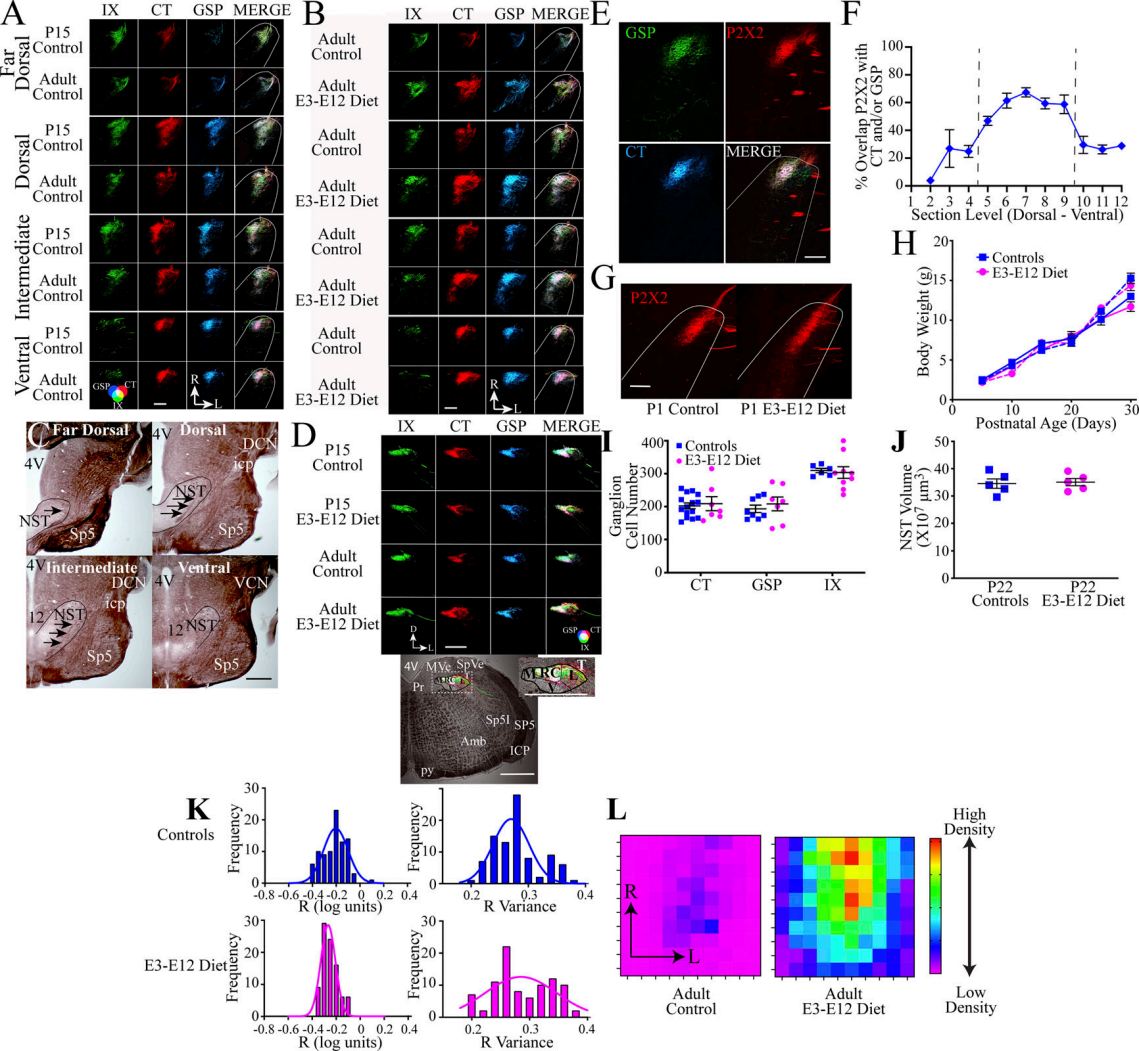
**Expanded photomicrographs of terminal fields in the NST, validation of P2X2**^**+**^ **as a proxy for CT and GSP labels, animal, and gustatory structural indices, and dLGN measurements. (A and B)** Horizontal sections in the four dorsal–ventral zones of the IX (green), CT (red), GSP (blue), and the merged image (MERGE) terminal fields in representative sections of P15 and adult control mice and in adult control and adult E3–E12 diet mice (A) and in representative sections adult control and adult E3–E12 diet mice (B). Refer to the color guide in the lower portion of A for overlap color shown in the merged images in A and B. Scale bars = 200 µm. R, rostral; L, lateral. The photomicrographs for the dorsal zones in Fig. S1, A and B are duplicated from Fig. 1, C and H, respectively. **(C)** Myelin-stained images of horizontal sections in an adult control mouse through the four dorsal–ventral zones of the NST. The NST is outlined in black, and arrows point to the solitary tract. 4V, fourth ventricle; Sp5, spinal trigeminal nucleus, DCN, dorsal cochlear nucleus; VCN, ventral cochlear nucleus; icp, inferior cerebellar peduncle; 12, hypoglossal nucleus. Scale bar = 1 mm. **(D)** Coronal sections of the brainstem showing terminal fields of the IX, CT, GSP, and the merged image in a P15 and adult control and adult E3–E12 diet mice. The lowest panel shows a low-power transmitted image of the right hemifield of the brainstem in a coronal section that contains terminal field labels in an adult control mouse. 4V, fourth ventricle; MVe, medial vestibular nucleus; SpVe, spinal vestibular nucleus; Sp5I, spinal trigeminal nucleus, interpolar; Sp5, spinal trigeminal nucleus; ICP, inferior cerebellar peduncle; AMB, nucleus ambiguous; and py, pyramidal tract. The NST is outlined by the dark lines, with the subdivisions labeled as M, medial; RC, rostral central; L, lateral; and V, ventral. Scale bar = 500 µm. Three animals/group were examined for gustatory terminal fields in coronal sections. **(E)** Photomicrographs of GSP and CT terminal fields and P2X2^+^ labeling in an adult control mouse. The MERGE image shows all fields together. Scale bar = 200 µm and the color wheel in the panel of the CT shows the overlapping colors. **(F)** The percent of overlap the P2X2^+^ labeled field has with the GSP and/or the CT terminal fields from the most dorsal to ventral consecutive sections in which there is CT or GSP terminal field labels. The vertical lines denote the range of sections used for calculating the volumes of the P2X2^+^ label as the proxy. Seven adult control mice were used to examine the overlaps of terminal fields with P2X2^+^ labeling. **(G)** Photomicrographs of the horizontal section containing P2X2^+^ labels in a P1 control and E3–E12 mouse. The white outline denotes the borders of the NST. The orientation is as shown in A and B. Scale bar = 50 µm. **(H)** Mean (± SEM) body weight of male and female controls and E3–E12 diet mice for the first 30 d postnatal. *n* for P5, P10, P15, P20, P25, and P30 controls and E3–E12 diet mice are 9, 10; 20, 16; 20, 20; 20, 16; 19, 16; and 17, 15, respectively. **(I)** Number of ganglion cells in the geniculate ganglion when the CT (*n* = 15, controls; *n* = 7, E3–E12 diet) or GSP (*n* = 8, controls; *n* = 7, E3–E12 diet) was labeled, or in the petrosal ganglion when the IX (*n* = 6, controls; *n* = 9, E3–E12 diet) was labeled. **(J)** The volume of the right hemifield of the NST in P20 controls (*n* = 5) and P20 E3–E12 (*n* = 5) mice. **(K)** Left: Quantification of R-distributions (R = log_10_ [inputs from ipsilateral/inputs from contralateral eye]; Torborg and Feller, 2004) in controls (top left) and E3–E12 diet mice (bottom left). The right two panels show the variance distributions of the R values for controls (top right) and E3–E12 diet mice (bottom right). **(L)** Heatmaps of putative CT synapses in the rostral NST in adult controls (left) and E3–E12 diet mice (right). The densities within each of the 10 × 10 grids (21.5 × 21.5 μm) represent the average density of CT labels in close apposition to PSD95 labels within each group. The density for each grid is normalized to the grid with the highest density found across all animals. The grid with the highest density is shown as red and occurs in approximately the center of the adult E3–E12 diet heatmap. R, rostral; L, lateral. One observation/animal was obtained and analyzed for data shown in F and H–J.

We found large-scale decreases in terminal field volumes of the chorda tympani (CT) and greater superficial petrosal (GSP) nerves from postnatal day 20 (P20) to P30, while no significant age-related change in terminal field volumes occurred throughout the development of the glossopharyngeal nerve (IX; Fig. 1, C–G; blue symbols and lines; Fig. S1, A and C). The amount of terminal field change of the CT and GSP was substantial (up to 61 × 10^5^ µm^3^): CT and GSP terminal field volumes decreased by 37% and by 58%, respectively, from P20 to P30 (Fig. 1, C, E, and F). By contrast, the terminal field size for the IX changed by only 5% from P15 to adulthood (Fig. 1, C and D), which differs from our finding that the rat terminal field volume also decreased with age (Mangold and Hill, 2008). There was no age-related change in terminal field volumes of the overlap among all three fields from P20–P30 (Fig. 1 G).

In contrast to controls, terminal fields of offspring of mothers fed a sodium-restricted diet from 3 to 12 d after conception (E3–E12 diet mice) underwent an age-related increase in CT and GSP terminal field volumes (75% and 25%, respectively) during the normal period of developmental pruning (Fig. 1, D–H; magenta symbols and lines; Fig. S1, B and C).

We then asked when terminal field volumes of the two diet-related groups initially diverged from each other. Beginning at P25, the CT and GSP terminal field volumes in E3–E12 diet mice and controls significantly diverged from each other, leading to field sizes in E3–E12 diet mice being approximately 2× greater than in controls by P30 (Fig. 1, E, F, and H). We also looked for group differences at P15 and at adulthood within well-defined subdivisions of the mouse NST, as defined in coronal sections (Bartel and Finger, 2013; Ganchrow et al., 2014), and found qualitatively lesser labels in both the rostral–central and in the lateral subdivisions of the NST for the CT and GSP terminal fields in adult controls (Fig. S1 D).

Because we were unable to reliably label all three nerves in mice younger than P15 due to surgical and anatomical limitations, we used a mouse reporter strain for the purinergic receptor, P2X2, as a proxy to visualize gustatory (CT and GSP) terminal fields (Breza and Travers, 2016; Kim et al., 2020). To validate the use of this reporter line as a proxy for CT and GSP labels, we identified that the P2X2^+^ label in P40 mice was collocated with at least 50% of the CT and/or GSP label in the intermediate and ventral sections of the NST (Fig. S1, E and F) and determined that P2X2^+^ labeling in P40 control and E3–E12 mice mirrored the diet-related differences found for CT and GSP terminal field labels (Fig. 1, E, F, and I). These reporter mice were then used to show that P2X2^+^-labeled terminal field size increased from P1 to P10 for both groups but with marginally less P2X2^+^ labeling in E3–E12 diet mice compared with age-matched controls (Fig. 1 I). Finally, we did not observe obvious differences in the spatial organization of the P2X2^+^ labeled field at P1, indicating that the pathfinding of gustatory afferents soon after they reached their NST targets (Zhang and Ashwell, 2001a) was not disrupted (Fig. S1 G). We find these fundamental findings to be quite remarkable because the period of the dietary sodium restriction is over before the peripheral gustatory system is assembled (Mistretta and Liu, 2006; Patel and Krimm, 2010) and before the appearance of circuits conveying taste information to the brain (Zhang and Ashwell, 2001a; Fig. 1 B).

Interestingly, the early dietary manipulation had no effect on body weights, the number of geniculate ganglion neurons (cell somas of the CT and GSP), number of petrosal ganglion neurons (cell somas of the IX), or size of the NST (Fig. S1, H–J).

We then asked if the early dietary effects are expressed globally by studying the developing mouse retinogeniculate system, in which binocular terminal field rearrangements occur during the first postnatal week (Godement et al., 1984). Using surgical and statistical procedures common to studies of retinogeniculate development in mice (Torborg and Feller, 2004; Ziburkus and Guido, 2006), we found no differences in the distributions in retinogeniculate inputs between adult control and adult E3–E12 diet mice (Fig. 1 J and Fig. S1, K and L). Thus, the early dietary manipulation, which had such a profound influence on circuit development in the gustatory system, did not universally alter sensory systems.

### The expanded terminal field in E3–E12 diet mice is accompanied by increases in putative synapses

We then asked if the enlarged terminal fields in E3–E12 diet mice are accompanied by more synapses. To examine this, we first compared the volume of postsynaptic densities (PSD95^+^ immunolabeling) in the rostral NST and then compared the amount of association between PSD95^+^ labeling with CT-labeled terminals. The amount of PSD95^+^ labeling in adult E3–E12 diet mice was 57% more than in adult controls, indicating that more synapses (from gustatory and non-gustatory inputs) occurred in this region due to the early dietary manipulation (Fig. 1, K and L). Moreover, the number of CT-labeled terminals associated with the PSD95^+^ label in this region was 4.7× more in adult E3–E12 mice compared with adult controls (Fig. 1 M), providing evidence that the age- and diet-related differences seen in terminal field sizes (Fig. 1, C–G) represent corresponding synaptic inputs into their target, postsynaptic neurons. Finally, we found that the density patterns of putative CT synapses were similarly located in the NST but with greater densities in E3–E12 diet mice than in controls (Fig. S1 L).

### Early dietary sodium restriction does not affect functional development of peripheral nerve taste responses but selectively alters taste-related behaviors

Neural activity plays a critical role in the sculpting of developing sensory systems (Chen and Regehr, 2000; Hooks and Chen, 2006; Katz and Shatz, 1996; Ziburkus and Guido, 2006). To test if the pruning period of terminal fields occurs concurrently with functional development, we focused on the CT because of its dramatic increased responsivity to salts postnatally driven by the functional development of the epithelial sodium channel (ENaC; Hendricks et al., 2000; Hill and Bour, 1985; Sun et al., 2017). We tested ages when sodium salt sensitivities of the CT are low (P15–P17), when they dynamically increase to near adult levels (P22–P27), and when responses are mature (>P40; adulthood; Ferrell et al., 1981; Hill and Almli, 1980; Hill and Bour, 1985; Hill et al., 1982; Yamada, 1980).

**Figure 2.**
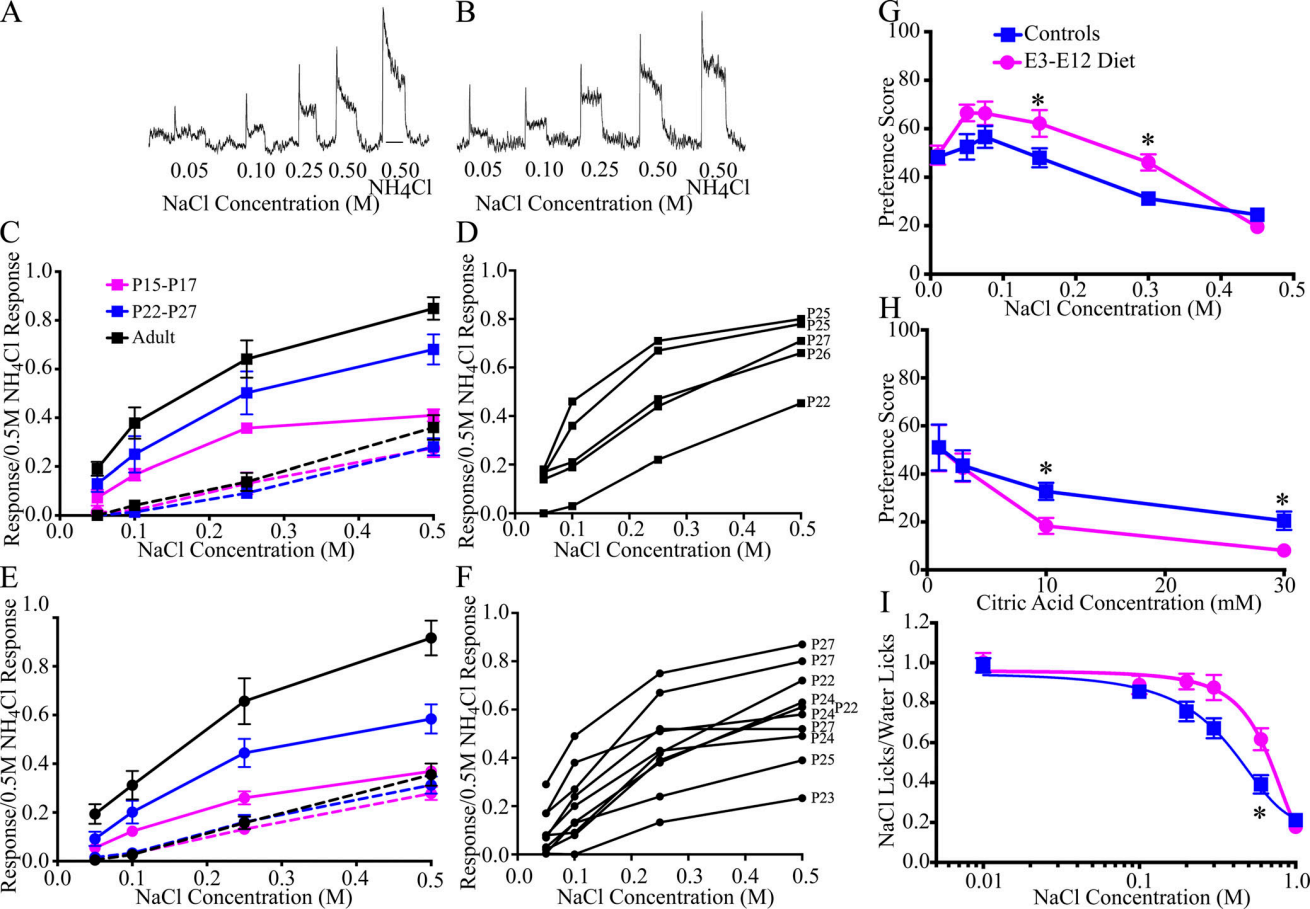
**Early dietary sodium restriction does not affect functional development of peripheral taste responses but selectively alters taste-related behaviors. (A and B)** Integrated taste responses from the CT to a concentration series of NaCl and 0.5 M NH_4_Cl in a P15 (A) and an adult (B) control mouse. The line drawn under the 0.5 M NH_4_Cl response in A denotes 20 s. **(C and E)** Relative taste responses in P15–17, P22–27, and adult controls (C; *n* = 5, 5, and 4/age group, respectively) and E3–E12 diet mice (E; *n* = 6, 5, and 4/age group, respectively) before (solid lines) and after (dashed lines) lingual application of amiloride. **(D and F)** Relative taste responses of individual control (D) and E3–E12 diet mice (F) to a concentration series of NaCl before lingual application of amiloride. The respective age of the mouse is listed adjacent to the response to 0.5 M NaCl. **(G and H)** Two-bottle preference tests in adult control (*n* = 5) and E3–E12 diet mice (*n* = 6) to a concentration series of NaCl (G) and citric acid (H). **(I)** Brief-access taste tests in adult control (*n* = 9) and E3–E12 diet mice (*n* = 12) to a concentration series of NaCl. For all dietary- and age-group comparisons, statistical comparisons were multiple, unpaired *t* tests using the Holm–Sidak method for multiple comparisons. Data in C, E, and G–I are shown as mean ± SEM. * denotes P < 0.05. **(G)** 0.15 M, P = 0.01; 0.3 M, P = 0.007. **(H)** 10 mM, P = 0.01; 30 mM, P = 0.015. **(I)** P = 0.007. In experiments where stable taste responses were recorded (maximum of three full series), relative responses were averaged across trials. Two-bottle taste responses were from one trial for each concentration/stimulus. Short-term access responses were averaged over the last 2 d of testing.

**Figure S2.**
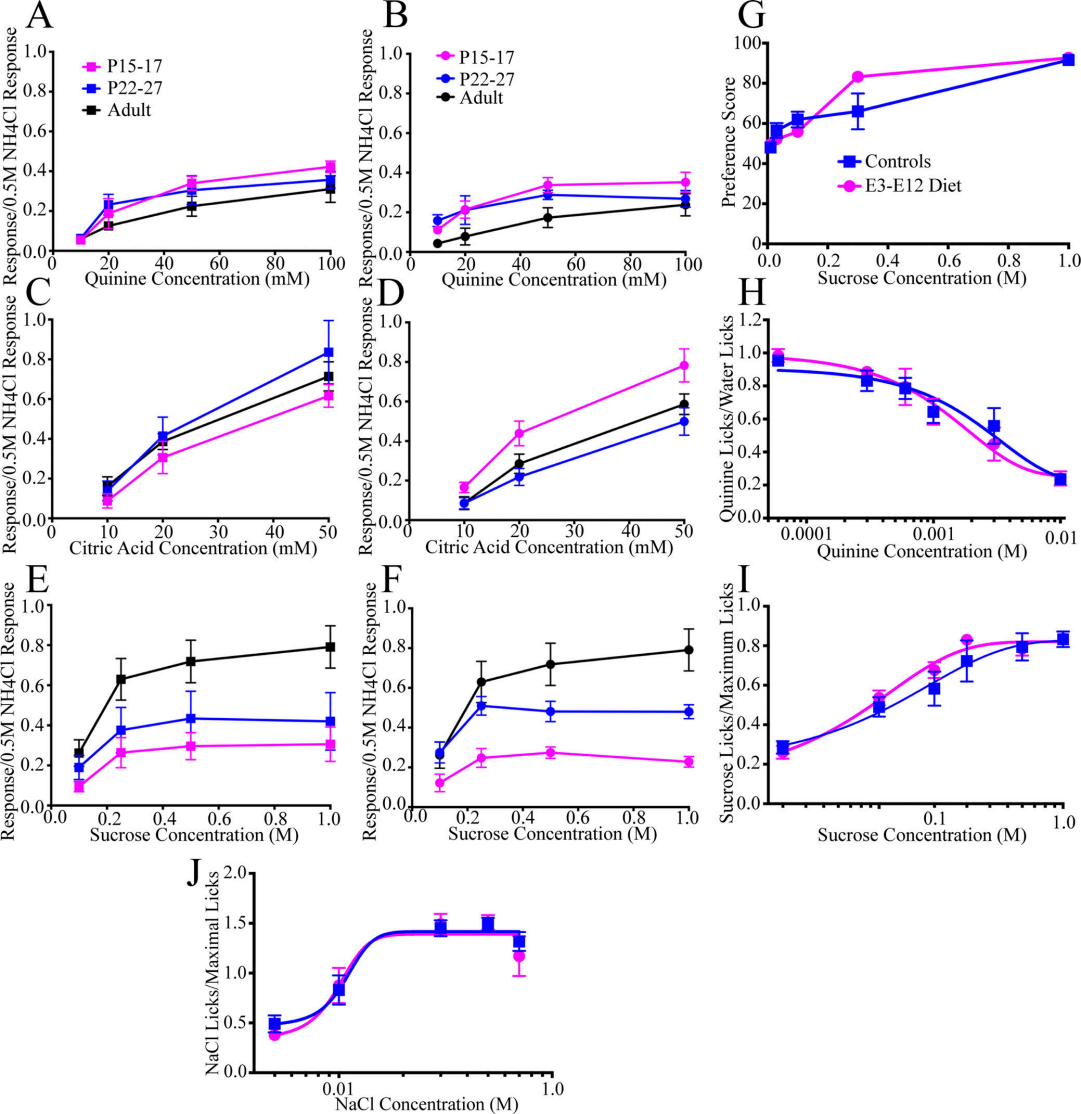
**Neurophysiological taste responses and behavioral measures where controls and E3–E12 diet mice were similar. (A–F)** CT responses to a concentration series of quinine hydrochloride in controls (A) and E3–E12 (B) mice, to a concentration series of citric acid in controls (C) and E3–E12 (D) mice, and to a concentration series sucrose in controls (E) and E3–E12 (F) mice. **(G)** Two-bottle preferences to a concentration series of sucrose in controls and E3–E12 mice. **(H and I)** Short-term access number of licks to a concentration series of (H) quinine hydrochloride and (I) sucrose in controls and E3–E12 diet mice. **(J)** Brief-access taste test in adult control (*n* = 9) and E3–E12 diet mice (*n* = 12) to a concentration series of NaCl after acute sodium depletion. Data are expressed as mean ± SEM. For all dietary-group comparisons, statistical comparisons were multiple, unpaired *t* tests using the Holm–Sidak method for multiple comparisons. No significant differences (P > 0.05) were found for any measures.

The relative taste response to NaCl in control mice increased dramatically from P15 to adulthood, with the largest change occurring between P22 and P27 (Fig. 2, A–D). Moreover, amiloride (an ENaC channel blocker; Benos, 1982) was effective in nearly eliminating the age-related increase in NaCl responses in controls (Fig. 2 C; compare dashed and solid lines). We found that the same developmental pattern of taste responses to NaCl also occurred in the CT of E3–E12 diet mice (Fig. 2, E and F). Moreover, no diet-related group differences in their CT responses were apparent to a concentration series of quinine, citric acid, or sucrose (Fig. S2, A–F). Thus, while age-related changes in functional taste responses likely impact pruning of terminal fields in controls, it likely does not account for the inability of E3–E12 diet mice to prune terminal fields.

Although peripheral taste function appears unaffected by our dietary manipulation, there may be behavioral diet-related effects that occur due to changes in central circuits. We conducted an array of behavioral taste testing that is often done as an initial survey in assessing taste sensibilities. They assessed relatively long-term preferences and aversions to a concentration series of NaCl, citric acid, sucrose, and quinine hydrochloride (48 h two-bottle taste preference tests), short-term preferences and aversions to a concentration series of NaCl, sucrose, and quinine (brief-access taste tests; Spector and Glendinning, 2009), and sodium-depletion induced salt appetite to a concentration series of NaCl (acute salt deprivation; Denton, 1969). Of the many tests, we found relatively few diet-related differences (Fig. 2, G–I; and Fig. S2, A–J). They were as follows: E3–E12 diet mice consumed relatively more mid-concentrations of NaCl than did controls (Fig. 2 G) and avoided relatively high concentrations of citric acid (Fig. 2 H). Oddly, while both groups showed aversive responses to citric acid, the E3–E12 diet mice found it more aversive than controls (Fig. 2 H). In short-access tests, E3–E12 diet mice failed to decrease the number of licks to relatively high concentrations of NaCl as controls (Fig. 2 I). It should be noted that all tests are relatively gross in their assessment of taste sensibilities and require more refined testing, including taste threshold and discrimination to fully assess the influence of altered pruning programs on behavioral outcomes. Nonetheless, there is evidence that behavioral consequences follow the large diet-related differences in the synaptic inputs to the first central relay in the gustatory system.

### Terminal field volume changes are accompanied by age- and diet-related differences in microglia densities in the NST, which are established as early as E9.5

To begin examining mechanisms responsible for the age- and diet-related differences in terminal field volumes, we asked if microglia play a role in shaping terminal fields of taste neurons. We chose to focus on microglia because of related work done on the development of the dLGN (Schafer et al., 2012; Stevens et al., 2007); however, we realize that mechanisms involving other cell types and molecular/biochemical pathways also play a role in pruning (Bjartmar et al., 2006; Chung et al., 2013; Shatz, 2009).

**Figure 3.**
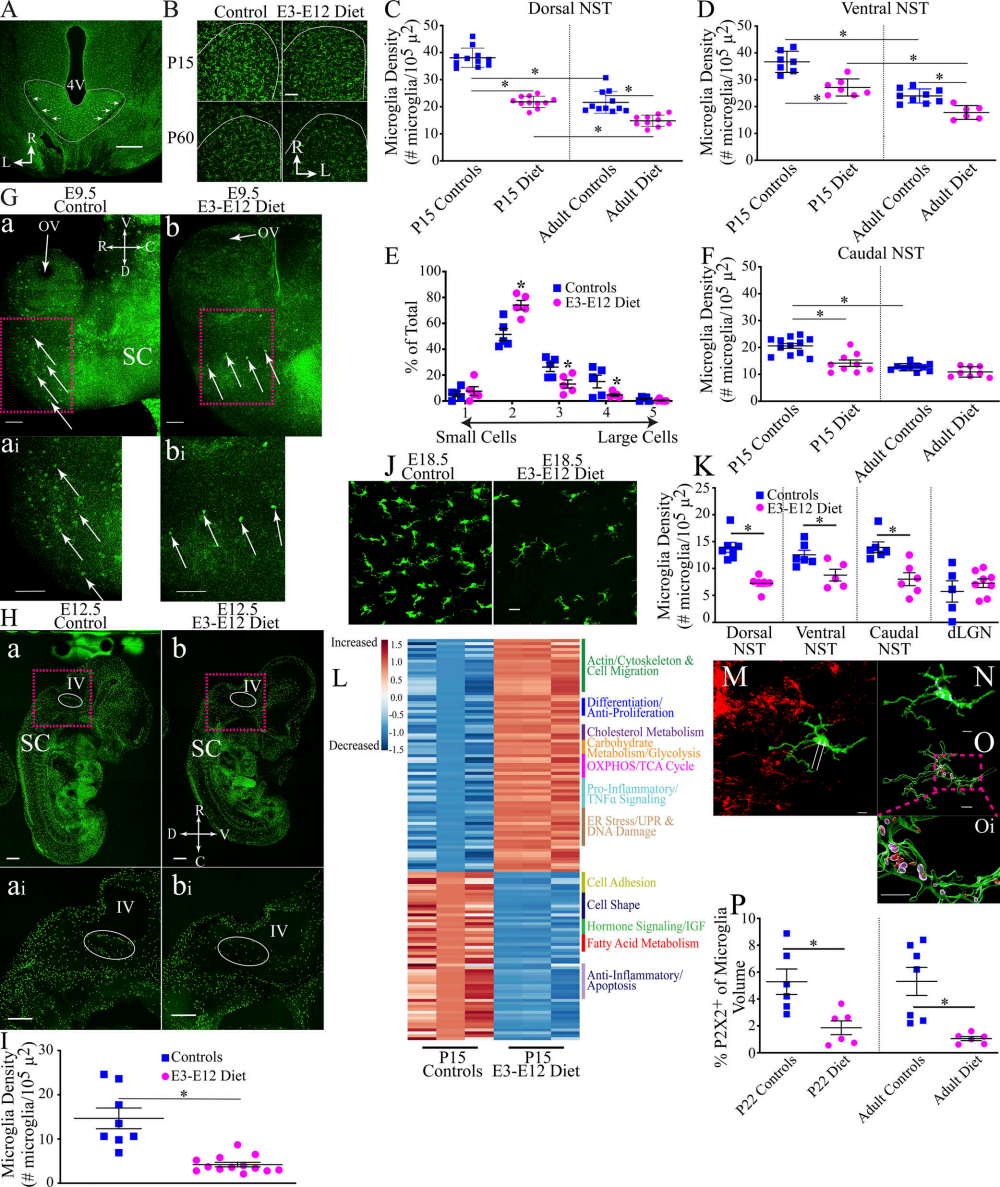
**Age- and diet-related differences in microglia densities in the rostral NST begin at E9.5 and extend into adulthood and have functional and molecular differences that may contribute to terminal field development and maintenance. (A)** Horizontal sections through a P15 control brainstem showing the NST to be rich in microglia. White lines show the border of the NST and arrows point to the solitary tract. Scale bar = 500 µm. R, rostral; L, lateral. **(B)** Microglia in a P15 and an adult (P60) control and E3–E12 mouse NST (white lines show the rostral NST border). Scale bar = 200 µm. R, rostral; L, lateral. **(C and D)** Microglia densities of individual animals and their means in the (C) dorsal and (D) ventral zones of the rostral NST. The number of animals analyzed for each group in the dorsal and ventral zones, respectively, are as follows: P15 controls (*n* = 11, 7); adult controls (*n* = 11, 9); P15 E3–E12 diet mice (*n* = 11, 7); and adult E3–E12 diet mice (*n* = 10, 6). **(E)** Percent of total microglia in the rostral NST in P22 control (*n* = 5) and E3–E12 (*n* = 5) mice scored in each of five categories to assess microglia morphology and lysosome activity. Microglia in controls are larger with fewer dendrites than E3–E12 diet mice. **(F)** Microglia densities of individual animals and their means in the caudal zone of the NST in P15 controls (*n* = 12); adult controls (*n* = 10); P15 E3–E12 diet mice (*n* = 9); and adult E3–E12 diet mice (*n* = 8). **(G, a and b)** Photomicrographs of images through the whole anterior portion of an E9.5 control (a) and E9.5 E3–E12 diet embryo (b). Arrows point to microglia in these CX3CR1 reporter mice. Inset panels (a i and b i) show higher magnifications of the respective area in the brain denoted by the magenta rectangles. The number of embryos examined were four controls and six E3–E12 diet mice. Scale bars = 100 µm. OV, optic vesicle; SC, spinal cord; V, ventral; D, dorsal; R, rostral; C, caudal. **(H)** Photomicrographs of images of an E12.5 control embryo (a and a i) and an E9.5 E3–E12 diet embryo (b and b i). Inset panels show higher magnifications of the respective area in the brain denoted by the magenta rectangle. The oval outlined in white denotes the location of the medulla. 8 controls and 13 E3–E12 diet animals were examined at E12.5. Scale bars = 500 µm. SC, spinal cord; V, ventral; D, dorsal; R, rostral; C, caudal. **(I)** Microglia densities of individual E12.5 embryos and their means in the medulla of the NST in controls (*n* = 8) and E3–E12 diet mice (*n* = 13). **(J)** Photomicrographs of microglia in the dorsal zone of the rostral NST in an E18.5 control (left panel) and E3–E12 diet (right panel) mouse. Scale bar = 20 µm. **(K)** Microglia densities of individual E18.5 embryos and their means in the dorsal and ventral zones of the rostral NST, the caudal NST, and the dLGN. The number of animals analyzed for each group in the four brain areas are: dorsal NST: E18.5 controls (*n* = 7), E3–E12 diet (*n* = 6); ventral NST: E18.5 controls (*n* = 7), E3–E12 diet (*n* = 5); caudal NST: E18.5 controls (*n* = 6), E3–E12 diet (*n* = 6); dLGN: E18.5 controls (*n* = 5), E3–E12 diet (*n* = 8). **(L)** Heat map showing upregulation (increased) or downregulation (decreased) of genes from microglia in P15 E3–E12 diet mice compared with P15 controls. The functional categories are shown to the right of the heat maps. Data were obtained from three independent replicates where the NST was isolated from six E3–E12 diet mice and six control mice/replicate, all at P15. **(M)** Photomicrograph of a microglia cell (green) within the P2X2^+^ terminal field. Arrows point to engulfed material (yellow) by the microglia. **(N)** Higher magnification of only the microglia in M showing the engulfed P2X2^+^ label (yellow) and CD68^+^ (blue) label denoting lysosomes. **(O)** 3D rendering of the microglia with the engulfed P2X2^+^ label (red) and lysosomes (transparent purple). **(O i)** Higher magnification of 3D-rendered microglia within the magenta rectangle shown in O. Scale bars for M–O = 3 µm. **(P)** Percent of large cell microglia occupied by engulfed P2X2^+^ label in P15 controls and E3–E12 diet mice (*n* = 6, 6, respectively) and adult controls and E3–E12 diet mice (*n* = 7, 6, respectively). Data from the three regions of interest in the NST were summed and analyzed for each animal. For all dietary and age group comparisons, statistical comparisons were multiple, unpaired *t* tests using the Holm–Sidak method for multiple comparisons. Data in C–F, I, K, and P are shown as mean ± SEM. * denotes P < 0.05. **(C)** P15 control versus adult control, P = 0.0001; P15 E3–E12 diet versus adult E3 = E12 diet, P = 0.0001; P15 control versus P15 E3–E12 diet, P = 0.0001; adult control versus adult E3–E12 diet, P = 0.0001. **(D)** P15 control versus adult control, P = 0.0001; P15 E3–E12 diet versus adult E3 = E12 diet, P = 0.0001; P15 control versus P15 E3–E12 diet, P = 0.0003; adult control versus adult E3–E12 diet, P = 0.0006. **(E)** Category 2, P = 0.01; category 3, P = 0.009; category 4, P = 0.02. **(F)** P15 control versus adult control, P = 0.0001; P15 control versus P15 E3–E12 diet, P = 0.0003. **(I)** P = 0.004. **(K)** Dorsal NST, P = 0.0001; ventral NST, P = 0.02; caudal NST, P = 0.004. **(P)** P15 controls versus P15 E3–E12, P = 0.01; adult controls versus adult E3–E12, P = 0.003. Apart from the data shown in L and P, only one observation/animal was made. Embryonic atlases (Allen Mouse Brain Atlas, 2004; Chen et al., 2017) enabled the identification of the medulla in E12.5 embryos.

The NST is rich in microglia (Fig. 3 A), and activation of microglia occurs in the rostral NST following sectioning of the CT in mice (Bartel, 2012; Bartel and Finger, 2013). Thus, microglia play a demonstrated injury-induced functional role in the NST where it receives gustatory inputs. We initially surveyed the densities and morphologies of microglia in the rostral NST during normal development. There was a higher density of microglia in the rostral NST of P15 control mice compared with adult controls (Fig. 3 B). In controls, the density of microglia in the dorsal and ventral zones of the NST decreased with age by ∼73% and 54%, respectively (Fig. 3, C and D), which is similarly described in developing rats (Riquier and Sollars, 2017). E3–E12 diet mice also showed age-related decreases in the density of microglia in the NST: a 47% and 50% difference in the dorsal and ventral zones, respectively (Fig. 3, B–D). However, and most importantly for this study, E3–E12 diet mice had at least a 33% lower density of microglia than in age-matched controls for both dorsal and ventral NST zones (Fig. 3, B–D).

**Figure S3.**
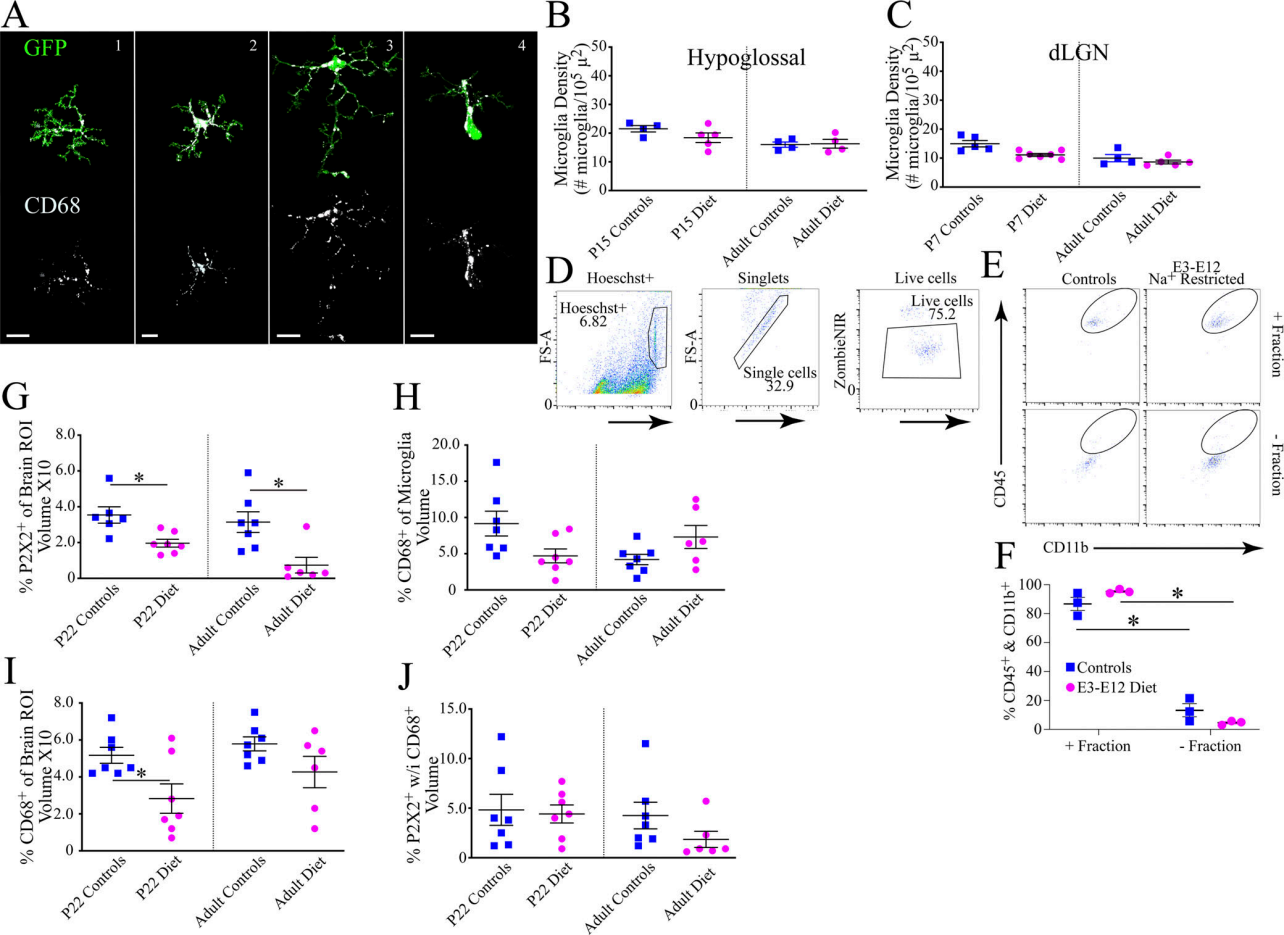
**Microglia activity categorization, densities in unaffected brain regions, isolation of microglia, and measures of engulfment of P2X2**^**+**^ **terminals and CD68**^**+**^**. (A)** Examples of the four categories in microglia. The top panels show examples of microglia categorized into each of the four categories. The bottom panels show the CD68^+^ label for each microglia example. Scale bars = 10 µm. Images of single microglia were extracted from confocal stack images of multiple microglia through Imaris software (Oxford Instruments). A black background was used to allow visualization of CD68^+^ labeling (white). Suitable examples of microglia representing category 5 could not be found in tissue imaged at higher magnifications used to provide clear images because they represent <1% of the total microglia. **(B and C)** Microglia densities of control and E3–E12 diet mice in the hypoglossal nucleus (B) and dLGN (control—P15, *n* = 4; adult, *n* = 4; E3–E12 diet—P15, *n* = 5; adult, *n* = 4; C). Note that measures were taken from P7 mice for lateral geniculate nucleus measurements to match the normal pruning period of this circuit (Schafer et al., 2012; Stevens et al., 2007). **(D and E)** Gating strategy (D) and representative plots (E) of microglia purity before RNA-seq. **(F)** Percent of CD45^+^ cells that are also CD11b^+^ in the fraction used for analyses (+ fraction) and negative fraction in samples from P15 controls (*n* = 3) and E3–E12 diet mice (*n* = 3). **(G)** Percent of P2X2^+^ label engulfed by microglia normalized to the volume of the brain sections analyzed in P15 controls and E3–E12 diet mice (*n* = 6, 7, respectively) and adult controls and E3–E12 diet mice (*n* = 7, 6, respectively). **(H)** Percent of large microglia occupied by CD68^+^ label in P15 controls and E3–E12 diet mice (*n* = 7, 7, respectively) and adult controls and E3–E12 diet mice (*n* = 7, 6, respectively). **(I)** Percent of CD68^+^ in microglia normalized to the volume of the brain sections analyzed in P15 controls and E3–E12 diet mice (*n* = 7, 7, respectively) and adult controls and E3–E12 diet mice (*n* = 7, 6, respectively). **(J)** Percent of P2X2^+^ colocalized with CD68^+^ in activated microglia in P15 controls and E3–E12 diet mice (*n* = 6, 7, respectively) and adult controls and E3–E12 diet mice (*n* = 7, 6, respectively). For all dietary group comparisons, statistical comparisons were multiple, unpaired *t* tests using the Holm–Sidak method for multiple comparisons. * denotes P < 0.05. **(F)** +Fraction versus −Fraction for controls, P = 0.0001; +Fraction versus −Fraction for E3–E12 mice, P = 0.0001. **(G)** P22 controls versus P22 E3–E12 diet, P = 0.007. **(H)** Adult controls versus adult E3–E12 diet, P = 0.008. **(I)** P22 control versus E3–E12 diet, P = 0.02. One observation/animal was obtained and analyzed for data shown in B, C, and G–J. For F, NSTs were pooled from six littermates with respect to treatment so that each biological replicate is representative of one litter. In total, three litters were used from control and from E3–E12 diet mice. Data in B, C, and F–J are shown as mean ± SEM.

To extend these findings, we assessed microglia morphology/lysosomal activity in the NST of P22 mice (an age when pruning of CT and GSP terminals occurs in controls; Fig. 1 E) by using a categorization scheme previously described (Schafer et al., 2012). Assignment of each microglia into one of the five categories was based on microglia morphology and amount of cluster of differentiation 68 positive (CD68^+^) immunostaining (lysosome-associated membrane protein; Fig. S3 A). There was a shift in the proportion of type 2 to types 3 and 4 microglia between dietary groups. E3–E12 diet mice had a significantly higher percentage (44%) of microglia in stage 2 (smaller cells with more extensive dendrites) and a significantly lower percentage in stages 3 (50%) and 4 (67%; larger globus-shaped microglia with fewer dendrites) compared with controls (Fig. 3 E and Fig. S3 A).

To see if age- and diet-related changes in microglia densities occurred globally, we analyzed microglia in three other brain areas—the caudal NST, where vagal inputs project (Contreras et al., 1982), in the hypoglossal nucleus, a motor nucleus located medial to the ventral zone of the NST that matures structurally from E7 to P28 (Kanjhan et al., 2016), and in the dLGN where axons from both eyes initially make overlapping inputs (Schafer et al., 2012). There were no age- or diet-related differences in the hypoglossal nucleus and dLGN; however, similar developmental changes in microglia densities noted for the gustatory zone of the NST were also present in the caudal NST (Fig. 3 F and Fig. S3, B and C). This indicates that age- and diet-related alterations in microglia densities also occur in areas where circuits make their inputs into the caudal NST but are not universally found throughout the CNS. We also note the impressively large numbers and densities of microglia in the rostral NST compared with other brain structures (Fig. 3, C, D, and F; and Fig. S3, B and C), which is consistent with the observed diversity in microglia numbers/densities throughout the brain (De Biase et al., 2017; Grabert et al., 2016).

These findings were extended by asking if the dietary group–related differences occur in microglia densities during embryonic development. We examined brains of embryos at E9.5, when microglia first colonize the brain from the yolk sac (Ginhoux et al., 2010), at E12.5, when the rudimentary medulla is formed (Chen et al., 2017; Zhang and Ashwell, 2001b), and finally, at E18.5, when the NST receives the initial projections from gustatory nerves (Zhang and Ashwell, 2001a).

Surprisingly, there were very few (or no) microglia in the brains of E3–E12 diet mice at E9.5 compared with age-matched controls (Fig. 3 G), indicating that the dietary manipulation influences the initial migration of microglia progenitors into the brain. All four E9.5 control mice had similar amounts of microglia in the brain, whereas five of six E3–E12 diet mice had no microglia labeling in the brain. The pattern of fewer microglia in E3–E12 diet mice continues at E12.5 (Fig. 3, H and I) and E18.5 (Fig. 3, J and K). Fig. 3 J also shows that the shapes of microglia in the rostral NST are qualitatively different between the two groups by E18.5. Microglia in E18.5 control mice had a more globous appearance and with relatively fewer extended dendrites—often categorized as amoeboid (Kaur et al., 2017)—and the densities of microglia in E3–E12 diet mice were lower than in controls (49% and 33%, respectively for dorsal and ventral zones; Fig. 3 K). We extended the analyses of microglia densities at E18.5 to other brain regions: the caudal NST and the dLGN. Like the rostral areas of the NST, there were 43% fewer microglia in the caudal NST of E18.5 E3–E12 diet mice compared with controls (Fig. 3 K). No difference was seen in the dLGN (Fig. 3 K). In summary, dietary sodium restriction during a short period early postconception selectively impacts the colonization of microglia in some but not all embryonic brain structures, and diet-related differences in densities are sustained in the medulla/NST throughout pre- and postnatal development.

### Microglia in the NST of P15 E3–E12 diet mice show a dysregulated transcriptional signature

To see if the expression of genes in microglia in E3–E12 diet mice is affected just before their expected involvement in the stripping and/or clearance of gustatory terminals in controls, we isolated microglia from the rostral NST of P15 controls and E3–E12 diet mice and then analyzed magnetic-activated cell (MAC)–sorted microglia for transcriptional signatures through RNA sequencing (RNA-seq; Fig. S3, D–F).

By segregating the RNA-seq data by functional groups, there appears to be an upregulation of genes involved with cell adhesion, migration, and extracellular matrix organization in E3–E12 diet mice (see Fig. 3 L; and Tables S1, S2, and S3). There also seems to be a metabolic switch in E3–E12 diet mice compared with controls, particularly with a loss of lipid metabolism (e.g., fatty acid metabolism) and an upregulation of genes necessary for glycolysis in E3–E12 diet mice (Fig. 3 L). Moreover, there was an upregulation of the oxidative phosphorylation system, the proinflammatory class of genes related to tumor necrosis factor alpha (TNFα) signaling, and signs of ER stress and DNA damage along with downregulation of anti-inflammatory gene families and insulin-like growth factor signaling in E3–E12 diet mice (Fig. 3 L). These differences in transcriptional signatures may underly a variety of functional/structural abnormalities in the NST that include synaptic stabilization and plasticity (Bergman and Ben-Shachar, 2016; Kaur et al., 2017), which could contribute to the altered development of gustatory terminal fields in E3–E12 diet mice.

### Microglia prune presynaptic material in both controls and E3–E12 diet mice but dramatically more pruning occurs in controls

To establish a functional relationship between microglia and terminal field refinement, we assessed the engulfment of presynaptic processes by microglia similar to that used in studies of the mouse dLGN and barrel cortex (Gunner et al., 2019; Norris et al., 2018; Schafer et al., 2012). We chose to study mice at P22 and at P40 (adult), which are ages when refinement of the CT and GSP terminal fields in controls are in the dynamic (P22) and steady-state phases (P40; Fig. 1, E and F). We examined areas that are medial, lateral, and caudal to the densest (core) region of the terminal field (Fig. 1, C and H; and Fig. S1, A and B) in the horizontal section for each animal in which the P2X2^+^ label corresponds most to CT and GSP labels (see Fig. S1, E and F). Due to the large diet- and age-related differences in the total amount of microglia labels, we chose to not use the total volume of microglia as the normalization factor for engulfment. Instead, we chose two different measures for normalization that did not vary among groups. We first normalized the amount of engulfment to the total volume of activated microglia (Schafer et al., 2012) in categories 2–4 (Fig. S3 A). We then normalized the total amount of P2X2^+^ label within microglia to the volume of the brain sampled, as done for our terminal field measurements (Fig. 1, D–G).

Terminal field label was found in microglia in all four groups (Fig. 3, M–P); however, the amount was dependent on the diet and not age. The amounts of P2X2^+^ label in activated microglia of P22 and adult E3–E12 diet mice were ∼36% and 20%, respectively, of that in age-matched controls. By contrast, the amounts of P2X2^+^ label in P22 and adult E3–E12 diet mice were ∼57% and 23% of age-matched controls, respectively, when the entire region of the brain sections was analyzed (Fig. 3 P and Fig. S3 G). Results from P22 controls are similar to those reported in studies of age-related pruning in the dLGN (Schafer et al., 2012), ∼5% label in NST microglia compared to 9% in dLGN microglia. Notably, there were no age-related differences between P22 and adult controls (Fig. 3 P and Fig. S3 G), indicating significant amounts of pruning by microglia continues into adulthood.

We further examined microglia for evidence of age- and diet-dependent differences in engulfment, defined previously as the amount of synaptic processes in microglia contained within a lysosomal marker (Schafer et al., 2012, 2014; Werneburg et al., 2020). There were no age or diet group differences in CD68^+^ labeling when assessed relative to activated microglia volume (Fig. S3 H) and only between P22 controls and diet mice when the entire brain regions of interest were analyzed—the amount of CD68^+^ label in P22 E3–E12 diet mice was 54% of the amount found in P22 controls (Fig. S3 I). The CD68^+^ results from P22 controls are similar to those reported in studies of age-related pruning in the dLGN (Schafer et al., 2012)—volumes of ∼10% CD68^+^ label in NST microglia and 15% in dLGN microglia. Finally, although all groups showed engulfment of the P2X2^+^ label, the amounts were relatively small. There were no age- or diet-related differences (Fig. S3 J); however, the comparison between adult controls and adult E3–E12 diet mice approached significance (P = 0.06).

The amount of engulfment in NST microglia is much less than that found in the dLGN (5% versus 9%; Norris et al., 2018; Schafer et al., 2012; Werneburg et al., 2020). However, there are major experimental factors and anatomical differences between these systems that hinder making direct comparisons for engulfment between the NST and dLGN. Experimentally, P2X2^+^ staining was used as a proxy for CT and GSP labels, representing 50–70% of the CT and/or GSP label (Fig. S1 F), and we chose to look early in the 10-d period when gustatory terminal fields are pruned (Fig. 1, E and F). This contrasts with the pathway-specific fluorescently tagged cholera toxin label used for engulfment assays in the retinogeniculate pathway and a relatively short period of binocular pruning in this pathway (2–3 d). Consequently, relatively more heterogeneous types and identities of P2X2^+^-labeled material were analyzed in NST microglia, and, conversely, some engulfed gustatory terminals may not have been detected because they were not labeled as P2X2^+^. Moreover, P2X2^+^ material may be internalized into NST microglia but contained within phagocytic compartments prior to degradation by lysosomes. Furthermore, the volume of the NST receiving gustatory inputs is ∼27× greater than the volume in the dLGN receiving binocular inputs, the terminal field label is less dense in the NST, and microglia are denser in the dLGN (Fig. 1, E and F; and Fig. 3, C and D). Taken together, and as noted by others, these factors point to limitations of using static imaging to extrapolate to a dynamic process such as engulfment (Gunner et al., 2019).

### Disruption of myeloid cells leads to a lack of pruning in normal development and activation of myeloid cell function induces pruning in E3–E12 mice

**Figure 4.**
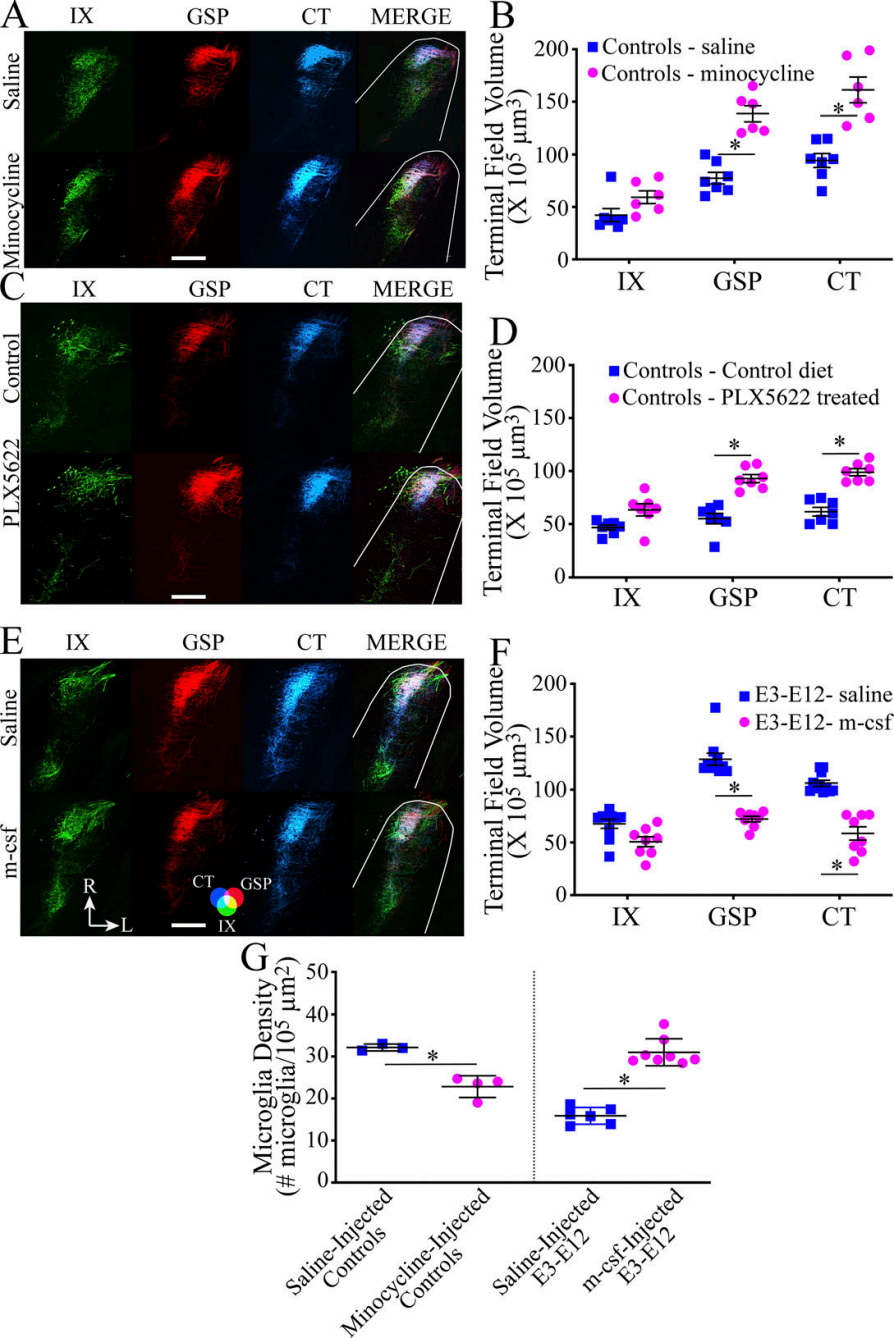
**Myeloid cells are involved in the pruning of terminal fields in control mice and can be activated to reversibly prune terminal fields in E3–E12 diet mice. (A)** Photomicrographs of horizontal sections through the intermediate zone of the NST showing IX (green), GSP (red), and CT (blue) terminal fields and the overlaps of all three nerves (MERGE) in control mice that were injected with saline or minocycline. **(B)** Terminal field volumes of the IX, GSP, and CT nerves in individual control mice (symbols) and their means injected with saline (*n* = 7) or with minocycline (*n* = 6) daily from P15 to P40. Minocycline-injected mice showed reduced pruning of GSP and CT terminals. **(C)** Horizontal sections through the intermediate zone of the NST showing IX, GSP, and CT terminal fields and the overlaps of all three nerves in control mice fed a control chow (control diet) or with PLX5622 added to the control diet (PLX5622 treated) from P18–P40. The photomicrographs are duplicated for the intermediate zone in Fig. S4 A. **(D)** Terminal field volumes of the IX, GSP, and CT nerves in control mice fed the control diet (*n* = 7) or the PLX5622-treated diet (*n* = 7). PLX5622-treated mice showed reduced pruning of GSP and CT terminals. **(E)** Horizontal sections through the intermediate zone NST showing IX, GSP, and CT terminal fields and the overlaps of all three nerves in E3–E12 diet mice who were injected with saline or m-csf. The photomicrographs are duplicated for the intermediate zone in Fig. S4 D. **(F)** Terminal field volumes of the IX, GSP, and CT nerves in E3–E12 diet mice and their means injected with saline (*n* = 10) or with m-csf (*n* = 8) every third day from P10 to P40. m-csf–injected mice showed a pruning of GSP and CT terminals. **(G)** Microglia densities and their means in the rostral NST in saline (*n* = 3) or minocycline-injected control mice (*n* = 4) and for saline- (*n* = 6) or m-csf–injected E3–E12 diet mice (*n* = 8). Refer to the color guide in the lower portion of E for colors shown in the merged images in A, C, and E. Scale bars = 200 µm. R, rostral; L, lateral shown in the lower portions of E. All statistical comparisons were multiple, unpaired *t* tests using the Holm–Sidak method for multiple comparisons. Data are shown as mean ± SEM. * denotes P < 0.05. **(B)** GSP, P = 0.00003; CT, P = 0.0004. **(D)** GSP, P = 0.000001; CT, P = 0.000001. **(F)** GSP, P = 000001; CT, P = 0.00006. **(G)** Minocycline, P = 0.002; m-csf, P = 0.007. One observation/animal was used for analyses.

**Figure S4.**
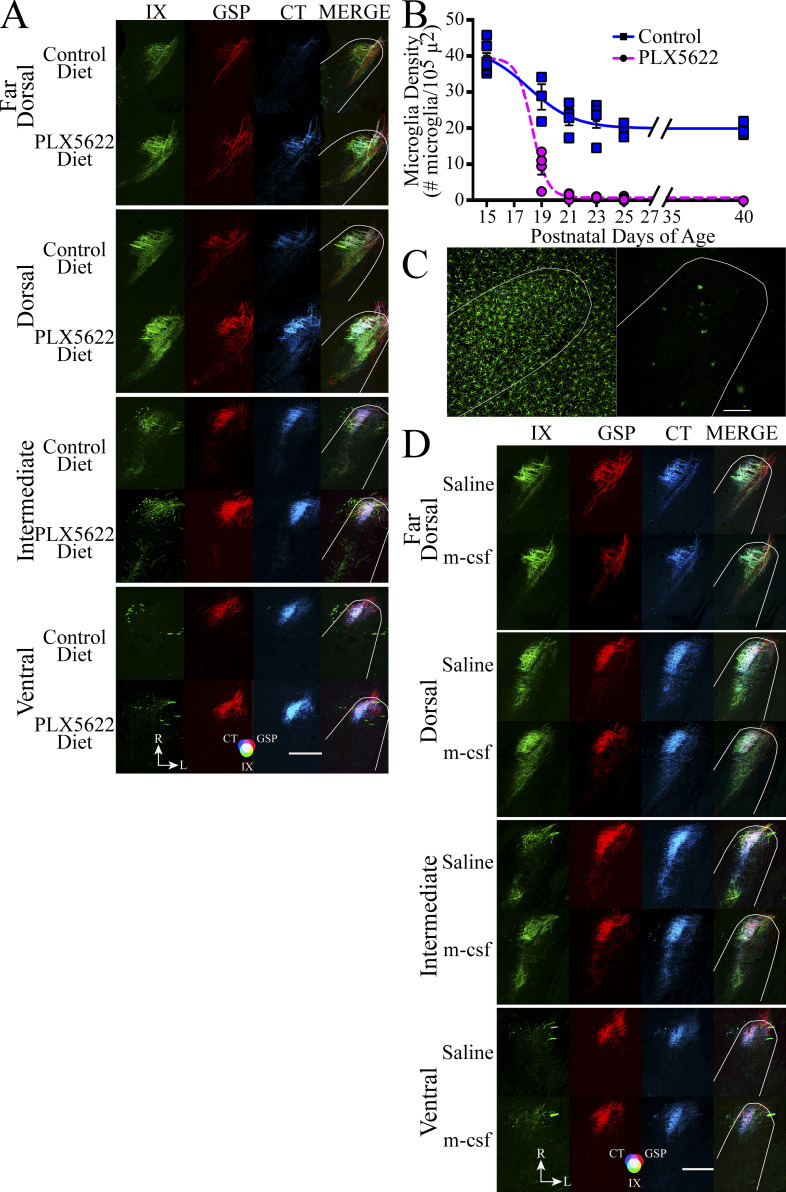
**Expanded photomicrographs of terminal fields in the NST in control mice treated with PLX5622 or E3–E12 diet mice treated with m-csf. (A)** Horizontal sections of labeled terminal fields of the IX, GSP, CT, and the merged image in the far dorsal, dorsal, intermediate, and ventral zones in control mice fed the control diet or the diet with PLX5622. The photomicrographs for the intermediate zone in Fig. S4 A are duplicated from Fig. 4 C. **(B)** Microglia densities (mean ± SEM) in P15–P40 mice fed the control diet or the diet containing PLX5622 from P18–P40. The numbers/group for controls and E3–E12 diet mice were P15 (7, 7, respectively), P19 (3, 4, respectively), P21 (6, 5, respectively), P23 (4, 3, respectively), P25 (3, 4, respectively), and P40 (7, 4, respectively). **(C)** Horizontal sections of P25 control mice fed the control diet (left) or the diet containing PLX5622 (right). The white outline denotes the rostral border of the NST. Scale bar = 100 µm. **(D)** Horizontal sections of labeled terminal fields of the IX, GSP, CT, and the merged image in the far dorsal, dorsal, intermediate, and ventral zones in E3–E12 diet mice injected with saline or with m-csf from P10–P40. Scale bars in A and D = 200 µm. The photomicrographs for the intermediate zone in Fig. S4 D are duplicated from Fig. 4 E.

We then asked if the plasticity of CT and GSP terminal fields can be induced in adulthood by manipulating myeloid cell function. We first pharmacologically treated control mice during the period of pruning with minocycline, which inhibits myeloid cell activation and has been used similarly in studies examining the pruning of developing retinogeniculate terminals (Schafer and Stevens, 2010). Injecting minocycline in control mice from P15–P40 resulted in an 80% and 70% larger GSP and CT terminal field volume, respectively, than in vehicle-treated controls. There was no effect on the IX terminal field (Fig. 4, A and B). We realize that there may be off-target effects of minocycline other than on microglia (Möller et al., 2016). Therefore, we used a more myeloid cell–specific method of inhibiting microglia function, which was to deplete microglia through the ingestion of rodent chow containing the colony-stimulating factor receptor 1 (CSFR1) antagonist, Plexxikon 5622 (PLX5622; Elmore et al., 2014). We found very similar results to those when control mice injected with minocycline: PLX5622-treated mice failed to prune CT and GSP terminal fields (Fig. 4, C and D; and Fig. S4 A). We also verified that the number of microglia was essentially eliminated within 3 d of feeding PLX5622 (Fig. S4, B and C). Collectively, these results show that the terminal field organization in adult controls can be induced to look like that in E3–12 diet mice by experimentally inhibiting myeloid cell function.

We then asked if the lifelong effects of the E3–E12 diet could be reversed in adulthood by activating myeloid cell function with macrophage colony–stimulating factor (m-csf; Döring et al., 2015). Indeed, m-csf was a potent factor that induced pruning of the GSP and CT terminal fields: terminal fields decreased by 77% and 80%, respectively, compared with vehicle-treated E3–E12 diet mice (Fig. 4, E and F; and Fig. S4 D). The IX was not altered with the m-csf injections (Fig. 4, E and F; and Fig. S4 D).

Curiously, significant decreases in the density of microglia in the rostral NST accompanied the lack of pruning in minocycline-injected mice, and conversely, significant increases in density of microglia in E3–E12 diet mice injected with m-csf accompanied the rescuing of pruning (Fig. 4 G).

### Multiple mechanisms likely contribute to the developmental pruning and maintenance of terminal fields in controls and are disrupted in E3–E12 diet mice

One of the mechanisms proposed to operate in the early postnatal pruning of binocular, retinogeniculate synapses is the classical complement cascade, which is driven by sequenced neuron/microglia interactions (Schafer et al., 2012; Stephan et al., 2012; Stevens et al., 2007). The central proteins in this cascade are C1q and C3 (Stephan et al., 2012), and deletion of the *C1q* or the *C3* gene results in the lack of synaptic pruning of retinogeniculate synapses in the dLGN during early postnatal development (Schafer et al., 2012; Stevens et al., 2007).

**Figure 5.**
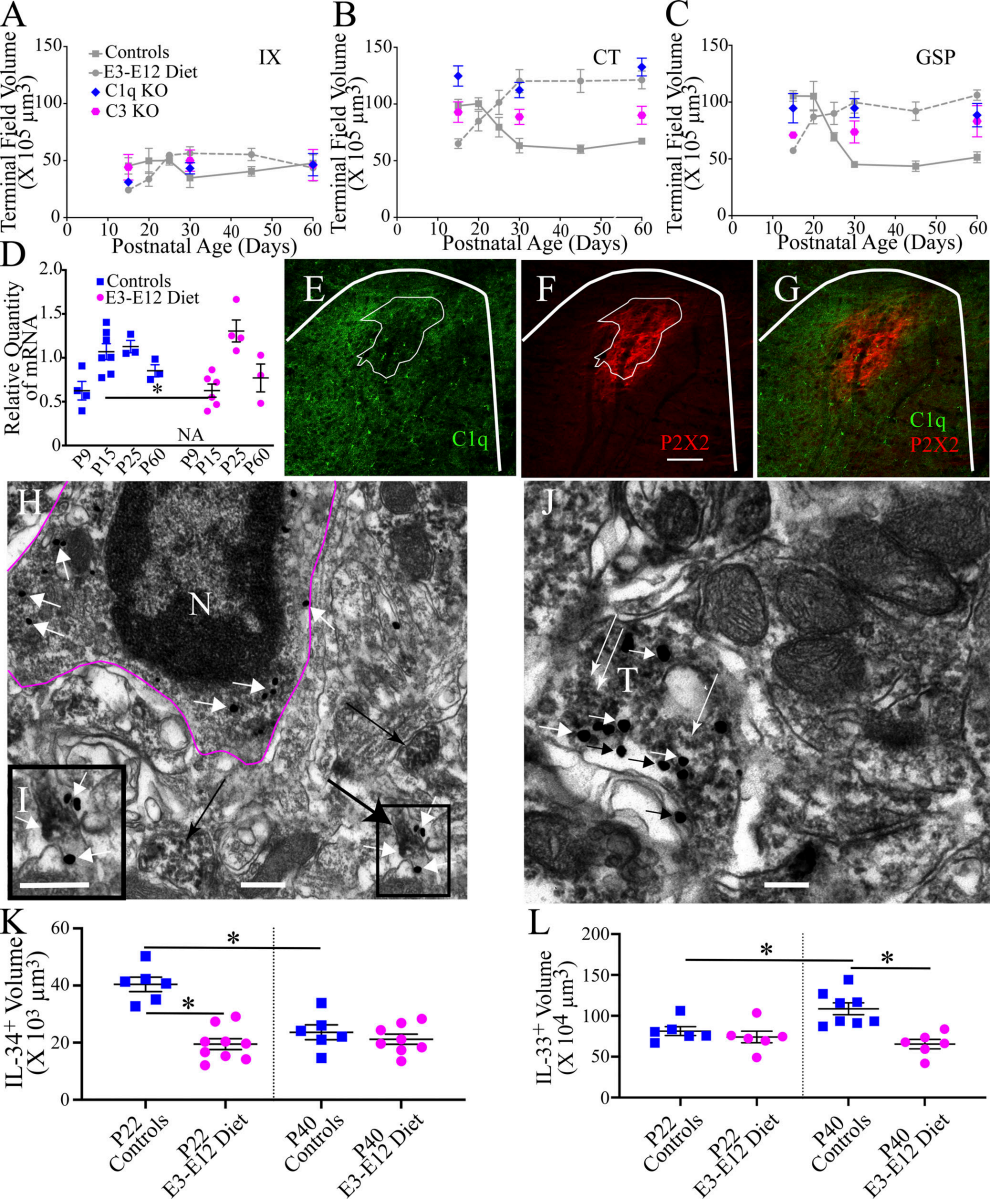
**The classical complement cascade participates in the pruning of terminal fields in control mice and may be disrupted in E3–E12 diet mice. (A–C)** Terminal field volumes of the IX, CT, and GSP in C1q and C3 knockout mice (colored symbols) at P15, P30, and adulthood (P60). They are shown with developmental terminal field data from controls and E3–E12 diet mice (gray lines and symbols) from Fig. 1, D–F. The number of C3 and C1q knockout animals are P15 (5, 4, respectively), P30 (5, 6, respectively), and P60 (5, 5, respectively). **(D)** qPCR data for C1qa during development in the geniculate ganglion in controls and in E3–E12 diet mice. Both groups show an age-dependent peak expression of C1qa, but the expression is delayed in E3–E12 diet mice compared with controls. Geniculate ganglia for two animals/group were pooled and analyzed as a single sample. The number of animals/groups are P9 controls (*n* = 4), P15 controls and E3–E12 diet (7, 6, respectively), P25 controls and E3–E12 diet (3, 4, respectively), and P60 controls and E3–E12 diet (3, 3, respectively). NA, not available. **(E and F)** Photomicrographs through the rostral NST of a control P25 mouse showing widespread C1q^+^ staining (E, green) and P2X2^+^ staining (F, red), indicating gustatory terminal field location and staining for both antibodies. The number of P15, P25, and adult control and E3–12 diet mice analyzed for C1q^+^ immunohistochemistry was *n* = 5/group. **(G)** The core region (densest P2X2^+^ staining) is outlined by thin white lines in E and F, illustrating that the core gustatory projection is nearly devoid of C1q. Thicker white bars denote the rostral border of the NST. Scale bar in F = 200 µm. **(H–J)** Electron micrographs from the rostral NST in a P25-d control mouse immunostained for C1q and P2X2. **(H)** The magenta line outlines a microglia cell (N, nucleus) that contains immunopositive gold staining for C1q. The box outlined in the lower right portion of the micrograph shows a P2X2^+^ stained terminal with C1q^+^ labeling on and near the terminal. **(I)** An enlarged view of the box outlined in the lower right panel of H. **(J)** A P2X2^+^ terminal (T) labeled with numerous C1q^+^ gold profiles. Short black and white arrows point to C1q^+^ gold profiles in H–J. The large black arrow in H points to the P2X2^+^ terminal shown in I. The thin black arrow in H points to a P2X2^+^ terminal, but without C1q^+^ staining. Scale bars in H–J = 350 nm. **(K)** IL-34^+^ staining in the rostral NST in P22 control and E3–E12 diet mice (*n* = 5, 9, respectively) and in P40 control and E3–E12 diet mice (*n* = 6, 8, respectively). **(L)** IL-33^+^ staining in the rostral NST in P22 control and E3–E12 diet mice (*n* = 6, 6, respectively) and in P40 control and E3–E12 diet mice (*n* = 8, 6, respectively). For all dietary and age group comparisons, statistical comparisons were multiple, unpaired *t* tests using the Holm–Sidak method for multiple comparisons. Data in A–D, K, and L are shown as mean ± SEM. * denotes P < 0.05. Significant statistical comparisons for B are P15: E3–E12 diet mice with C1qKO, P = 0.003; P30: controls with C1qKO, P = 0.0005; and P60: controls with C1qKO, P = 0.0006; for C, they are P15: controls with C3KO, P = 0.0006 and E3–E12 diet mice with C1qKO, P = 0.01; P30: controls with C1qKO, P = 0.001 (also see Table S4). **(D)** P15 control versus P15 E3–E12 diet, P = 0.02. **(E–G)** C1q within microglia and located in the core region: P15 control versus adult control, P = 0.02; C1q within microglia but located within the surround region: P15 control versus adult control, P = 0.01; P15 E3–E12 diet versus adult E3–E12 diet, P = 0.008; C1q not within microglia but located in the core region: P15 control versus P15 E3–E12 diet, P = 0.01; C1q not within microglia and located in the surround region: P15 control versus P15 E3–E12 diet, P = 0.01. **(K)** P22 control versus P22 E3–E12 diet, P = 0.0001; P22 control versus P40 control, P = 0.0009. **(L)** P22 control versus P40 control, P = 0.01; P40 control versus P40 E3–E12 diet, P = 0.0008. One observation/animal was obtained and analyzed for data shown in A–C, K, and L.

We also found that deletion of the *C1q* or the *C3* gene resulted in the lack of pruning of gustatory inputs in the NST. IX, CT, and GSP terminal field volumes in C1q and C3 knockout mice did not alter throughout development by more than 15% (Fig. 5, A–C). This resulted in significant differences in mean terminal field sizes between controls and E3–E12 diet mice with C1q and C3 knockout mice for the GSP and CT at P15, P30, and P60 (adults; Table S4). No differences among means with the knockout mice occurred for the IX (Table S4). Importantly, the observation that terminal field sizes for the CT and GSP did not change (i.e., failure to prune) in C1q and C3 knockout mice is the critical finding here. Interpretation of significant differences among terminal field volumes with controls and E3–E12 diet mice is not straightforward because there are non-complement C1q-related processes occurring in the CNS (Thielens et al., 2017), and C3 is affected by multiple upstream proteins that are not complement related (Stephan et al., 2012).

We also examined the developmental expression of the three *C1q* chains (*C1qA*, *C1qB*, and *C1qC*) in the cell somas that make up the CT and GSP in the geniculate ganglion (see Fig. 1 A). All three *C1q* chains were upregulated in the geniculate ganglion in a developmentally dependent manner in control mice, with the peak expression at P15 (Fig. 5 D), the age just before terminal fields begin to decrease in size. Interestingly, a similar developmental-related pattern of expression occurred in E3–E12 diet mice. However, the age at which the peak expression of *C1q* in E3–E12 diet mice was later—at P25—than found for controls (Fig. 5 D). This developmental delay may be critical if C1q levels must be synchronized to when peripheral taste activity rapidly increases (see Fig. 2, A–F).

We next investigated if the C1q protein in the NST was expressed in a developmental- and diet-dependent manner. This was a difficult task because the level of C1q^+^ staining at all ages and in both dietary groups was widespread and particularly dense throughout the NST (see Fig. 5 E). We did discover, however, something very unexpected. The region of the densest terminal field label (i.e., the core) was largely devoid of C1q^+^ labeling, whereas the region surrounding the core showed C1q^+^ labeling in all age and dietary groups (Fig. 5, E–G). This is consistent with our observations that the region surrounding the core is what is pruned the most in the CT and GSP terminal fields of controls but not as much in E3–E12 diet mice. We found no differences in overall positive immunostaining for C1q in the rostral NST between dietary groups at P15, P25, or in adulthood (Table S5). However, we did find greater densities of C1q^+^ staining within microglia, the primary source of C1q (Fonseca et al., 2017), in the core, and in the surrounding areas in P15 compared with adults in controls (Table S5). Surprisingly, there was significantly more C1q^+^ staining outside of microglia (i.e., the surround) in P15 E3–E12 diet mice compared with P15 controls in both the core and surrounding regions (Table S5), indicating that the cellular machinery producing C1q in the NST is intact just before pruning begins in both dietary groups.

To validate the presence and localization of C1q at gustatory-related terminals in controls, we turned to experiments using double-label immunohistochemistry for electron microscopic examination. Fig. 5, H–J shows C1q in close apposition to some, but not all, P2X2^+^ terminals, indicating that they are tagged by the initiator protein for the classical complement cascade.

Although it appears that the expression levels of *C1q* cannot account for the lack of pruning in E3–E12 mice, alterations in other molecular mechanisms involved in remodeling central gustatory terminals may be altered. We chose to briefly examine the components of two candidate mechanisms—IL-34 and IL-33.

IL-34 is key for the development of myeloid cell differentiation and maintenance through its action on the CSFR1 (Easley-Neal et al., 2019). It is principally produced by neurons, and deletion of *Il34* results in reduced numbers of microglia in specific brain regions (Cahoy et al., 2008; Zeisel et al., 2015). Importantly, it appears to be a ligand for activation of CSFR1 along with m-csf. While m-csf is critical in establishing microglia in the embryonic brain, IL-34 expands and/or differentiates the progenitors of microglia postnatally in mice as well as plays a role in the maintenance of microglia in adulthood (Baghdadi et al., 2018; Easley-Neal et al., 2019; Wang et al., 2012). Interestingly, work with zebrafish shows that progenitors of microglia are attracted to brain regions containing IL-34 and its receptor (Wu et al., 2018).

**Figure S5.**
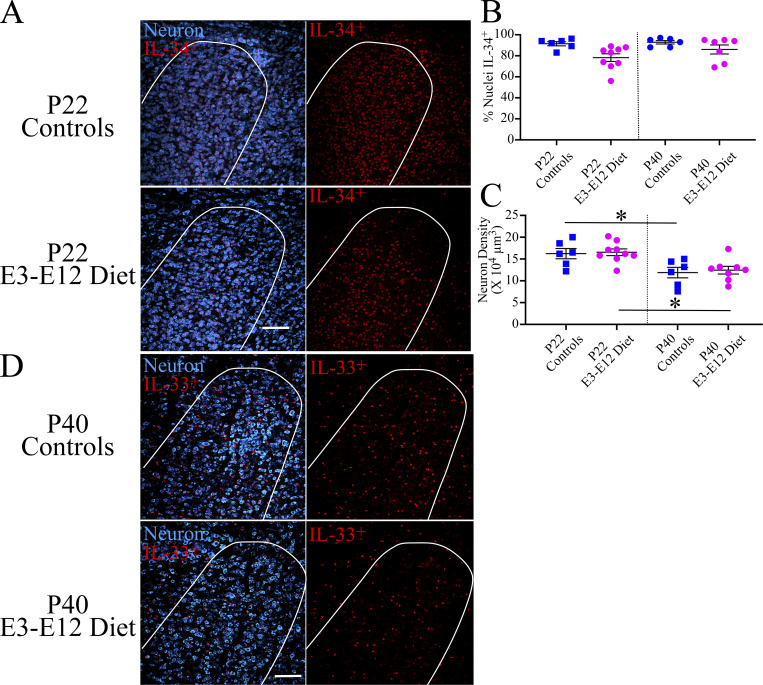
**IL-33 and IL-34 also show age- and diet-related differences in the rostral NST. (A)** Photomicrographs of an optical, horizontal section in the rostral NST (outlined in white) in a P22 control and E3–E12 mouse showing IL-34^+^ staining (red) in nuclei of NST neurons (blue; left panels) and only with IL-34^+^ staining. **(B)** Percent of NST neurons containing IL-34^+^ staining in neuronal nuclei in the rostral NST in P22 controls and E3–E12 diet (*n* = 6, 9, respectively) and in P40 control and E3–E12 diet mice (*n* = 6, 7, respectively). **(C)** NST neuron densities in the rostral NST in P22 controls and E3–E12 diet (*n* = 6, 9, respectively) and in P40 control and E3–E12 diet mice (*n* = 6, 7, respectively). **(D)** Photomicrographs of the rostral NST (outlined in white) in a P22 control and E3–E12 mouse showing IL-33^+^ staining (red) in nuclei of NST neurons (blue; left panels) and only with IL-33^+^ staining. For all dietary group comparisons, statistical comparisons were multiple, unpaired *t* tests using the Holm–Sidak method for multiple comparisons. **(C)** * denotes P = 0.02 and 0.003 for P22 versus adult for controls and E3–E12 diet mice, respectively. Scale bars in A and D = 100 µm.

Here, we examined the amount of Il-34^+^ staining in the rostral NST in P22 and P40 controls and E3–E12 diet mice and found that the amount (i.e., volume) of IL-34^+^ was 100% greater in P22 controls compared with P22 E3–E12 diet mice and 70% greater compared with P40 controls (Fig. 5 K). Similar amounts of IL-34^+^ staining were found between P22 and P40 E3–E12 diet mice and between P40 controls and E3–E12 diet mice (Fig. 5 K). Interestingly, the percentage of NST neurons containing IL-34^+^ staining was similar among all groups (Fig. S5, A and B), although the density of all neurons in the NST was greater in P22 control and E3–E12 diet compared with their P40 counterparts (Fig. S5 C). Collectively, this indicates that the amount of I-34^+^ staining in P22 controls was greater than in age-matched E3–E12 diet mice and not because of differences in neuron densities. That is, similar percentages of NST neurons (nearly 100%) produced IL-34 but the average production/neuron was greatest in P22 controls. While only speculative at this time, IL-34 may play a complementary and/or additional role in pruning terminal fields because the period of maximum labeling coincides with the peak of terminal field organization exclusively in controls. Moreover, its receptor is shared with m-csf, which was shown earlier to induce pruning in E3–E12 diet mice (Fig. 4, E and F).

In contrast to IL-34, IL-33 is produced primarily by astrocytes and is demonstrated to promote metabolic adaptation and synaptic engulfment through its receptor, ST2, in microglia during early development (He et al., 2022). We asked if IL-33^+^ staining was more pronounced in the NST of P22 and P40 controls compared with age-matched E3–E12 diet mice. What we found was surprising in that the amount of IL-33^+^ staining in the NST in P40 controls was significantly greater compared with the other three groups (Fig. 5 L and Fig. S5 D), suggesting that mechanisms, as indicated by others, exist throughout development to regulate cellular metabolism and to potentially influence pruning of terminal fields (Gadani et al., 2015; He et al., 2022).

## Discussion

We show here that the immune system plays a key role in the refinement and maintenance of central gustatory circuits during postnatal development, and these processes can be circumvented by events occurring before the peripheral gustatory system is assembled prenatally. That is, simply altering the mother’s dietary sodium during a limited and very early period of their offspring’s prenatal development arrests the normal neural/immune program(s) that sculpts gustatory circuit development much later in life. We believe that the decrease in dietary sodium during this early phase of embryonic development impacts the migration and function of microglial progenitors as they transition from the yolk sac to the embryonic brain, and aberrations in neuro-immune function are sustained throughout development, likely resulting from altered function of multiple molecular mechanisms.

### Microglia are active players in the pruning of gustatory terminal fields

Our results indicate that microglia engulf presumptive terminal fields in control mice when they are reorganizing. The process of pruning is similar to that described for the binocular pruning for the developing retinogeniculate pathway (Schafer et al., 2012; Stevens et al., 2007); however, the period of pruning is significantly later, more extensive, and longer in the gustatory system. The differences between visual and gustatory circuit refinement clearly illustrate the specialization of the resident microglia population for densities and function of the respective brain area undergoing synaptic remodeling. Indeed, this is a theme that we observed throughout development. Surprisingly, we also found that substantial amounts of pruning continue into adulthood in controls, but not for E3–E12 diet mice. The lack of pruning in adult E3–E12 diet mice results in an apparent excess of synaptic inputs onto NST neurons, which is consistent with previous neurophysiological and ultrastructural findings following lifelong dietary sodium restriction in rats (May et al., 2008; May and Hill, 2006; Vogt and Hill, 1993). Curiously, not all areas of the brain are affected by the E3–E12 dietary manipulation, and in fact, the retinogeniculate pathway appears untouched.

The discoveries found here further highlight the remarkable lifelong plasticity in peripheral and central gustatory pathways. In particular, the experimental manipulation of myeloid cell function not only leads to a loss of pruning (and presumed decreased engulfment) of CT and GSP terminals in adult control mice but also decreases the number and density of microglia in the rostral NST. Conversely, activating myeloid cell function at adulthood through the injection of m-csf not only prunes CT and GSP terminal fields in E3–E12 diet mice but also restores the number and density of microglia in the rostral NST to control levels. While some of the diet-related differences in terminal field development could simply be explained by corresponding differences in microglia densities, it is likely that other factors also account for the diet-related differences. Morphological and molecular indices show that the function of microglia, at least in the rostral NST, has been affected by dietary manipulation.

### Multiple molecular mechanisms are likely involved in pruning of CT and GSP terminal fields in controls, with some of them dysregulated in E3–E12 diet mice

One of our aims was to closely examine one of the molecular mechanisms operational in the normal pruning gustatory terminals and then test if the mechanism was altered in E3–E12 diet mice. We chose to study the classical complement cascade in detail because of its demonstrated role in pruning and developing retinogeniculate synapses (Schafer et al., 2012; Stevens et al., 2007). We found significant similarities between controls for the two sensory systems. They include (1) a failure to prune terminal fields and synapses in C1q knockout and C3 knockout mice, (2) the peak of C1qA, B, and C chain expression in primary afferent neuron cell bodies occurs just before the peak pruning period, (3) a close association of the initiator protein (C1q) with synapses from sensory inputs into the brain, and (4) regulation of pruning is coincident with increased sensory-driven activity. It is unlikely that dysregulation of the classical complement cascade occurred in E3–E12 diet mice because the development of taste activity in the CT was similar to controls, and the geniculate ganglion and NST produced similar amounts of C1q, although the peak production of C1q in the ganglion was developmentally delayed by 10 d. Additionally, the expression of at least 30 complement genes (e.g., C1q, CD11b, C3, and CxCR3) in the microglia of P15 mice was similar between groups, indicating that other functional and molecular alterations occurred in E3–E12 mice.

We emphasize here that the complement pathway is probably not the sole mechanism shaping developing taste circuits. Rather, just as in the developing retinogeniculate pathway (Bjartmar et al., 2006; Chung et al., 2013; Huh et al., 2000; Shatz, 2009), other pathways likely contribute to the refinement of gustatory circuits. In fact, we show here that IL-34 and IL-33 may play key roles in the sculpting and maintenance of these circuits through multiple processes and types of cells (e.g., neurons and astrocytes).

During normal development, IL-34 may act coincidently with the complement cascade to prune circuits by operating on the same or different circuits (i.e., circuits for different taste modalities and/or for different nerves). We found IL-34, which involves neuron-to-microglia signaling through the CSFR1 receptor (Wang et al., 2012) had maximal expression in control neurons at the peak of terminal field pruning and not in E3–E12 diet mice or in adult controls. IL-34 shares its receptor with m-csf and appears to provide complementary functions in activating CSFR1. Most notably, they play complementary roles in determining microglia numbers. M-csf may be critical in the initial colonization of the brain with microglia; however, both ligands are involved in the postnatal development and maintenance of these cells (Wang et al., 2012). Curiously, stimulation of the CSFR1 with m-csf in adult E3–E12 diet mice induced pruning of the gustatory terminal field (Fig. 4, E and F), perhaps mimicking what would normally involve IL-34.

Unexpectedly, we found that IL-33 was only overexpressed in adult controls compared with younger controls and in E3–E12 mice. Although it is not clear why this molecule, generated by astrocytes (He et al., 2022), is only overexpressed in adulthood, it may be required to maintain control terminal field sizes throughout adulthood. This is consistent with our observation that the default conformation without normal neural and/or immune function is the enlarged terminal field size seen in postnatal rats and mice. Evidence for this is supported by our previous studies in which the primary rodent transduction channel for sodium taste was deleted throughout development or in adulthood and in which competitive interactions among the CT, GSP, and IX were manipulated. All experimental manipulations led to the return of the field to its immature size and structure (Corson and Hill, 2011; Skyberg et al., 2017; Sun et al., 2017). Stated more simply, the normal molecular processes function to maintain smaller, mature terminal fields.

### The origins of the pruning program are set within the first few days of embryonic brain development

**Figure 6.**
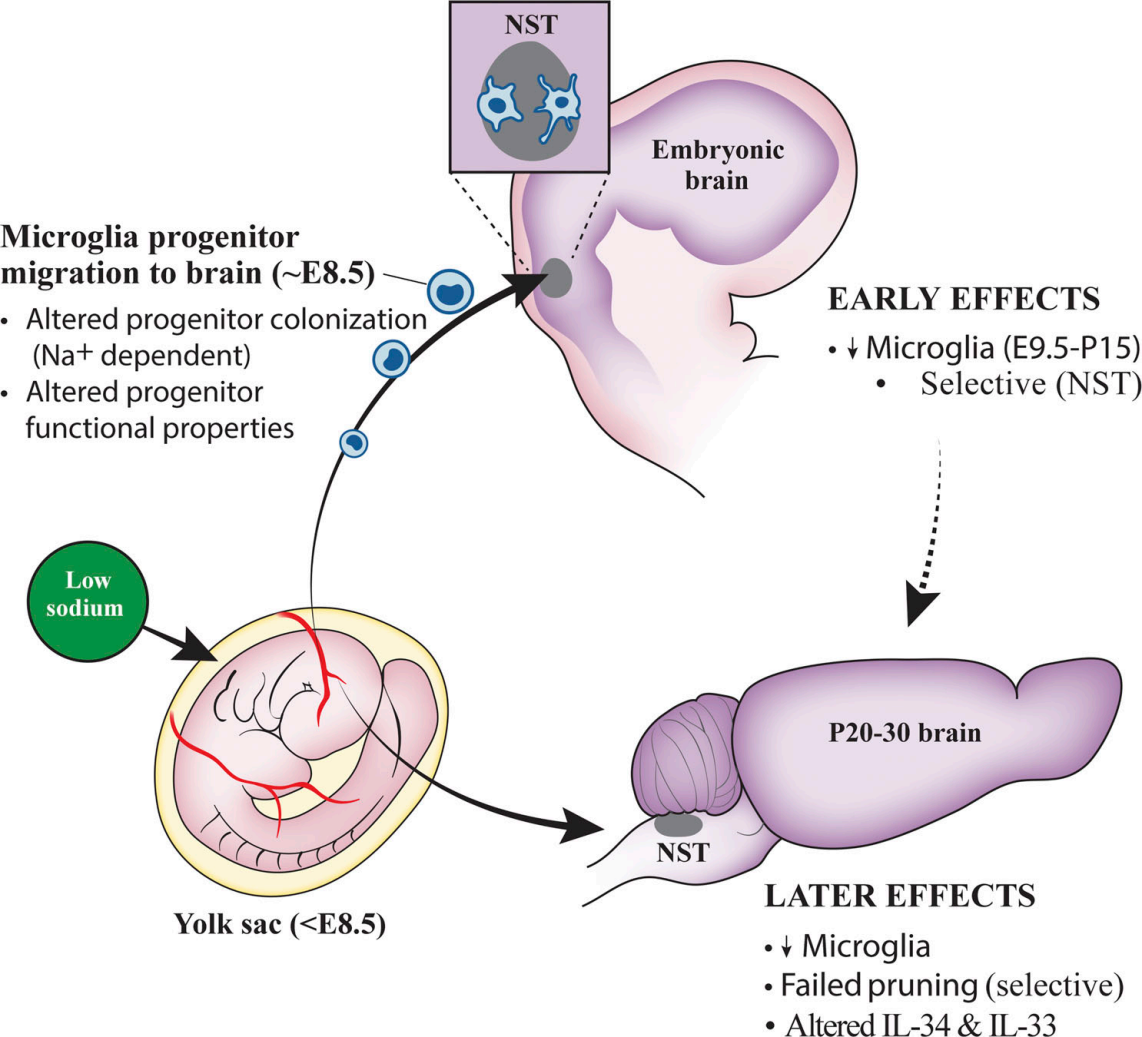
**Model of potential mechanism(s) of early prenatal dietary sodium restriction on microglia numbers and postnatal failure to prune gustatory terminals in the NST.** A maternal low-sodium diet impacts the environment of the embryo when microglia progenitors are transported into the emerging brain of the embryo (∼E8.5) by altering the cellular processes of the progenitors and/or compromising the number of progenitors that take up residence in the NST (solid arrow). This produces a decrease in microglia numbers. A later consequence (∼3–4 wk) of this altered early environment is decreased numbers of microglia and a failure to prune gustatory terminal fields in the NST (solid arrow). The dotted arrow denotes a direct link between the early and later effects, which could involve multiple molecular mechanisms for pruning and maintenance of terminal field integrity in adulthood (e.g., IL-34 and IL-33).

Perhaps our most interesting discovery is that significant decreases in microglia densities in E3–E12 diet mice begin as soon as microglia first colonize the brain and then have lifelong effects on central gustatory circuits. Fig. 6 depicts a possible scenario where simply altering the sodium content of the mother’s diet during the critical period when microglia progenitors migrate from the yolk sac to the emerging brain severely alters this migration. These early embryonic events subsequently lead to dramatically altered postnatal development of gustatory projections at the first central synaptic relay. Further support for this hypothesis comes from experiments done in rat—shifting the start of the dietary 9-d sodium-restriction period before E3–E12 or after E12 had no effect on the adult CT terminal field, indicating that the most sensitive period for this effect is somewhere around E6–E9 (Krimm and Hill, 1997). This is the age that was later shown to be when the first wave of yolk sac progenitor cells migrates into the early-developing brain (Ginhoux et al., 2010; Stremmel et al., 2018; Fig. 1 A). We hypothesize that low NaCl levels compromise sodium-dependent nutrient and protein transport systems in the placenta and the yolk sac (Holdsworth and Wilson, 1967; Yadgary et al., 2011), and this loss of function impacts differentiation and/or migration of these cells that take up residence in some brain areas including the NST (Fig. 6). This is consistent with the finding that genetically disrupting heart and blood circulation function from E9.5–E10.5 through a defect in the sodium-calcium exchanger 1 led to a lack of microglia migrating from the yolk sac to the brain (Ginhoux et al., 2010; Koushik et al., 2001).

We find it interesting that some but not all brain areas appear affected by this early environmental insult. The effects are not global because the hypoglossal nucleus and the dLGN did not have age- or diet-related microglia density differences. Conversely, we do not rule out that structures other than the NST may be affected. Such heterogeneity of brain areas impacted may be related to developmental factors such as when cohorts of yolk sac progenitors enter the brain, their migration to their final destinations, and/or local factors imposed by specific brain areas (e.g., m-csf and IL-34). Some of these factors have been implicated as directing heterogeneity of microglia structure and function in multiple basal ganglia nuclei (De Biase et al., 2017).

Future research may identify the mechanisms underlying the cellular and molecular processes that are affected by environmental influences during the critical period of microglia progenitor differentiation and migration into the embryonic brain. For example, other environmental events could mirror the effects of the low-sodium diet. We found that dietary protein restriction in rats starting in early embryonic development (beginning at E3) produced alterations in CT and GSP projections into the NST similar to that shown here (Thomas and Hill, 2008). Moreover, the effects may not be specific to central gustatory circuits—other central circuits are likely altered by the early embryonic environment. For example, circuits originating in the gut may be affected like the gustatory inputs because of our observed age- and diet-related alterations in microglia densities in the caudal portion of the NST. More generally, the effects of this early embryonic perturbation in immune function could have more wide-reaching roles in the genesis of developmental disorders and neurological diseases.

## Materials and methods

### Animals

Unless stated otherwise, all mice were group-housed, maintained on standard rodent chow and tap water, and weaned at P21. Male and female mice were used for all measures. There were no sex-related differences (P > 0.05) in groups large enough for statistical testing (i.e., *n* = 4 or larger/group).

#### Terminal field development in the NST

The development of terminal fields used control (C57Bl/6) and E3–E12 sodium-restricted mice (E3–E12 diet mice) at P15, P20, P25, P30, P45, and P60. Control mice were either purchased from Jackson Laboratories or were raised in the University of Virginia vivarium. E3–E12 diet mice were fed a low-sodium rodent diet (0.03% NaCl; MP Biomedicals) via their mothers from E3 to E12 and then returned to a standard rodent chow (0.3% NaCl; rodent diet, #8604; Harlan Teklad) throughout the remainder of their life. The mothers of these mice were also given distilled water (ad libitum) during the diet manipulation and then switched to tap water. Control mice were provided the standard rodent chow throughout their development. The numbers of mice/age for control and E3–E12 diet groups were, respectively, P15 = 5, 6; P20 = 6, 6; P25 = 5, 5; P30 = 5, 5; P45 = 4, 5; adult (>60 d) = 5, 5. P1 terminal fields were studied in five controls and five E3–E12 diet mice. The presence of a sperm plug was taken as E0.

#### Terminal field development in the dLGN

The terminal fields of retinal geniculate ganglion terminal fields in the dLGN were examined in three adult (>P60) controls and three adult E3–E12 diet mice.

#### P2X2 reporter mice were used to examine terminal fields in P40, P1, P5, and P10 mice

A knock-in P2X2-cre mouse line (Kim et al., 2020) was crossed with a cre-sensitive tdTomato reporter mouse (*B6.Cg-Gt(ROSA)26Sortm9(CAG-tdTomato)Hze/J*; #007909; Jackson Labs) to visualize P2X2 expression in the NST of P40 (controls, *n* = 3; E3–E12 diet, *n* = 4), P1 (controls, *n* = 5; E3–E12 diet, *n* = 5), P5 (controls, *n* = 5; E3–E12 diet, *n* = 6), and P10 (controls, *n* = 5; E3–E12 diet, *n* = 5) mice.

#### PSD95 and CT labeling in the NST

Combined PSD95 immunostaining and CT nerve labels were done in seven adult control and seven adult E3–E12 mice.

#### Taste responses from the CT

Neurophysiological taste responses were recorded from five, five, and four control mice aged P15–P17, P22–27 adult controls, respectively, and six, five, and four E3–E12 diet mice, respectively.

#### Taste-related behavioral tests

Two-bottle preference tests were conducted with five adult control and six E3–E12 diet mice and short-access taste tests were assessed in nine adult control and twelve adult E3–E12 diet mice.

#### Microglia density measures

The number of animals analyzed for each group in the dorsal and ventral zones, respectively, were as follows: P15 controls (*n* = 11, 7); adult controls (*n* = 11, 9); P15 E3–E12 diet mice (*n* = 11, 7); and adult E3–E12 diet mice (*n* = 10, 6). The number of animals in the caudal zone, hypoglossal nucleus, and dLGN was as follows: P15 controls (*n* = 12, 4, 5, respectively); adult controls (*n* = 10, 4, 4, respectively); P15 E3–E12 diet mice (*n* = 9, 5, 7, respectively); and adult E3–E12 diet mice (*n* = 8, 4, 5, respectively). Four E9.5 control embryos and six E3–E12 diet mice were examined for microglia, and densities were made from E12.5 controls (*n* = 8) and E3–E12 diet (*n* = 13) mice. At E18.5, microglia densities were from the dorsal and ventral zones of the rostral NST and the caudal NST in controls (*n* = 6) and E3–E12 diet (*n* = 6) mice and from the dLGN in controls (*n* = 5) and E3–E12 mice (*n* = 8).

#### Categorization of microglia by morphology and CD68^+^ staining

Microglia from five P22 control and five P22 E3–E12 diet mice were used to categorize microglia for differences in size and lysosome-related staining. The number of microglia analyzed/animal ranged from 82 to 110 in controls (mean ± SEM = 97.8 ± 1.2) and 36–104 in E3–E12 diet mice (76 ± 3.8).

#### RNA-seq samples

The NST was dissected bilaterally from six P15 control and six P15 E3–E12 diet mice. There were three independent replicates totaling 36 P15 mice.

#### Microglia engulfment measurements

Engulfment measurements were done in the rostral NST in P15 and P40 (adult) controls (*n* = 6, 7, respectively) and P15 and adult E3–E12 diet (*n* = 6, 6, respectively).

#### PLX5622, minocycline, and m-csf experiments

Littermates from three litters were weaned at P18 onto a control diet (*n* = 7) or the control diet containing PLX5622 (Opna Bio; *n* = 7). They were maintained on each diet to at least P40, which was when nerve labels were done to assess terminal field sizes. Tap water was available throughout the period. Body weights were taken every day. There was an initial 2 d during which body weights did not increase, but it was then followed by a normal increase in body weight throughout the period. There were no diet-related differences between males or females.

Littermates from two control litters were injected with physiological saline (*n* = 7) or with minocycline (*n* = 6; 50 mg/kg; i.p.; #M9511; Sigma Aldrich), daily from P15 to at least P40, which was when nerve labels were done to assess terminal field sizes.

Littermates from four E3–E12 diet mouse litters were injected with physiological saline (*n* = 10) or with m-csf (*n* = 8; 40 µg/kg body weight; i.p.; 576406; BioLegend) every third day from P15 to at least P40, which was when nerve labels were done to assess terminal field sizes.

#### C3 and C1q knockout mice

C3 knockout mice were obtained from Jackson Laboratories (029661; B6.129S4-*C3*^*tm1Crr*^/J) and terminal field labels were performed at P15, P30, and in adulthood, with five mice/group. C1q knockout mice were provided as a kind gift from Dr. Marina Botto (C1qA knockout mice; C57BL6 background) and terminal fields were obtained as for the C3 knockout mice.

#### C1q and P2X2 immunohistochemistry in the NST

The NST was examined for C1q and P2X2-positive immunohistochemical staining in three age groups—P15, P25, and P40. There were five controls and five E3–E12 diet mice in each age group. Additionally, four P18 controls were used for electron microscopic experiments.

#### Quantitative PCR (qPCR) analyses for C1qa in geniculate ganglion

Unfixed left and right geniculate ganglia from controls aged P9 (*n* = 8), P15 (*n* = 10), P25 (*n* = 6), and P60 (adults; *n* = 6), and from E3–E12 sodium-restricted mice aged P15 (*n* = 12), P25 (*n* = 10) and P60 (*n* = 6) were collected like that described for geniculate ganglion counts. For analyses, geniculate ganglia for two animals/group were pooled and analyzed as a single sample.

#### IL-34 and IL-33 immunohistochemistry in the NST

Control mice at P22 and P40 (*n* = 6, 6, respectively) and E3–E12 diet mice (*n* = 9, 8, respectively) were used to stain for IL-34, and control mice at P22 and P40 (*n* = 6, 8, respectively) and E3–E12 diet mice (*n* = 6, 6, respectively) were used to stain for IL-34.

### Methods detail

All experiments were approved by the University of Virginia Animal Care and Use Committee and followed guidelines set forth by the National Institutes of Health and the Society for Neuroscience. For all experiments, data analyses were performed blind to the experimental group. All experimental data were verified in at least three independent experiments.

### Nerve labels

The triple-label and perfusion procedures were performed according to methods detailed in a previous publication (Sun et al., 2015) and are described briefly here.

Mice were sedated with a 0.32 mg/kg injection of Domitor (medetomidine hydrochloride; Pfizer Animal Health; intramuscularly [I.M.]) and anesthetized with 40 mg/kg Ketaset (ketamine hydrochloride; Fort Dodge Animal Health; I.M.). A water-circulating heating pad was used to maintain body temperature. Mice were positioned in a non-traumatic head holder, and a ventral approach was taken to expose the GSP and CT nerves within the right tympanic bulla. The CT and GSP nerves were cut near and peripheral to the geniculate ganglion in the tympanic bulla and crystals of 3 kD tetramethylrhodamine dextran amine were applied to the proximal cut end of the GSP and crystals of 3 kD biotinylated dextran amine were applied to the proximal cut end of the CT. The IX was isolated medial to the tympanic bulla, cut peripheral to the petrosal ganglion, and placed on a small piece of parafilm. Crystals of 3 kD cascade blue dextran amine were applied to the proximal cut end of the IX nerve. A small amount of Kwik-Sil (World Precision Instruments, Inc.) was then placed over the cut end of the nerves to prevent crystals from diffusing from the site of the intended label. All dextran amine conjugates were purchased from Thermo Fisher Scientific. Animals were then injected with 5 mg/ml Antisedan (atipamezole hydrochloride; Pfizer Animal Health; I.M.) to promote the reversal of anesthesia. Following 48-h survival, animals were deeply anesthetized with urethane and transcardially perfused with Krebs–Henseleit buffer (pH 7.3), followed by 4% paraformaldehyde (pH 7.2).

### Eye injections

Following anesthesia, adult mice received intravitreal injections of cholera toxin B conjugated with either Alexa Fluor 488 or Alexa Fluor 555 (C34775 and C34776; Thermo Fisher Scientific, respectively) into each eye, respectively (1 μl/eye; 1 µg/μl). Mice recovered on a heating pad and were perfused 48 h later.

### Tissue preparation and analysis of NST and LGN terminal field sizes in postnatal mice

Brains were removed and postfixed, and the medulla was blocked and sectioned horizontally on a vibratome at 50 μm (Sun et al., 2017) for analyses of gustatory terminal fields. We chose to section tissue in the horizontal plane because it allows visualization of the entire rostral–caudal and medial–lateral extent of the terminal fields in the NST with the smallest number of sections (∼10 sections/mouse). However, some of the terminal fields were examined in coronal sections to examine labels in subnuclei of the NST (see Fig. S1 D). For LGN tissue preparation, brains were blocked and sectioned coronally through the thalamus.

Brainstem sections were then incubated for 1 h in PBS containing 0.2% Triton with 1:400 streptavidin Alexa Fluor 647 (Jackson ImmunoResearch Labs) and 1:400 rabbit anti-Cascade Blue (Cat# A-5760, RRID:AB_2536192) at room temperature. Streptavidin Alexa Fluor 647 was used to visualize the biotinylated dextran amine-labeled CT-positive terminals. Rabbit anti-Cascade Blue was used as a primary antibody to detect Cascade Blue–labeled IX terminal fields and was followed by a 1-h reaction with 1:400 donkey anti-rabbit Alexa Fluor 488 (Cat# 711-545-152, RRID:AB_2313584; Jackson ImmunoResearch Labs). This secondary antibody was used to visualize IX nerve terminals. Visualization of tetramethylrhodamine, which labeled GSP terminal fields, did not require further processing. Sections were mounted on slides and coverslipped with Vectashield Hardset Mounting Medium (Vector Laboratories). Thalamic sections were rinsed in PBS and mounted on slides as described for brainstem sections.

Terminal fields were imaged using a Nikon 80i microscope fitted with a Nikon C2 scanning system (Nikon Instruments, Inc.) and a 10× objective (Nikon, CFIPlanApo; NA = 0.45). The nerve labels were matched for the wavelengths of the three lasers in the system (argon laser: 488 nm, 10 mW, IX; DPSS laser: 561 nm, 10 mW, GSP; modulated diode laser: 638 nm, 20 mW, CT). Sequential optical sections were captured every 3 μm for each 50-μm section. Images were obtained with settings adjusted so that pixel intensities were near (but not at) saturation. A transmitted light image at 4× (Nikon PlanFluor; NA = 0.13) and at 10× was captured for every physical section containing the labeled terminal field. This permitted an accurate registration of dorsal to ventral brainstem sections among animals within and between groups using common brainstem landmarks (4×) and identification of NST borders (10×). Three sections through the thalamus that contained the most ipsilateral and contralateral projections were collected and analyzed for terminal field labels in the LGN.

Methods used to analyze gustatory terminal field volumes and densities were described previously in detail (Sun et al., 2015). Briefly, quantification of terminal field volume was achieved with ImageJ-based software. Each image stack was initially rotated so that the solitary tract was oriented vertically. The border of the NST was outlined for each physical section using the corresponding transmitted light image and the stack was then cropped to include only the NST. The IsoData thresholder algorithm from ImageJ was then applied to yield a binary image stack of the labeled pixels above the threshold. Particle analysis was then performed to quantify the pixel area above the threshold for each channel. Specifically, the number of pixels above the threshold for each optical section was summed by ImageJ, converted into the area by multiplying the number of pixels by pixel size (1.24 × 1.24 µm), and then multiplied by 3 µm (i.e., distance between optical sections) to determine the volume of each label in each physical section. Volumes from each physical section were summed to yield the total terminal field volume for each mouse. The resultant volume represents an unbiased experimenter measure of the amount of label. Additionally, the volume of colocalization between the terminal fields of two nerves (CT with GSP, GSP with IX, CT with IX) and among all three nerves (CT, GSP, and IX) was determined in a similar manner as described for each single label.

A similar method was used for analyses of terminal field labels in the LGN, which examined the amount of contralateral, ipsilateral, and overlapping input from the eyes.

### Geniculate and petrosal ganglion cell counts

The CT or the GSP nerve was labeled as described for the terminal field labeling procedure, with the exception that the 3 kD tetramethylrhodamine dextran was chosen as the only tracer because it did not require further processing for visualization. After cardiac perfusion, the brain was removed to gain access to the ganglia. Geniculate ganglia were then removed ventrally through the base of the skull with the aid of a surgical microscope. Petrosal ganglia were also labeled with the tetramethylrhodamine tracer by way of the IX. Following cardiac perfusion, petrosal ganglia were removed along with a portion of the IX, using the approach described for the nerve labeling technique. All ganglia were postfixed overnight. Each intact ganglion was mounted on a slide and imaged on a scanning laser confocal microscope. Serial 2-μm optical sections were taken throughout each ganglion and all labeled cells were counted with Neurolucida (MBF Bioscience).

### Measures of NST volumes

The transmitted light images (4×) taken of all sections in P20 control and E3–E12 diet mice were used to determine if the size of the NST differed between groups. The NST volume was measured using Neurolucida computer software (version 4.34; MBF Bioscience). To calculate volume, the area measurements from all the sections were summed and multiplied by the tissue thickness of 50 µm.

### P2X2 reporter mice used to examine terminal fields in P40, P1, P5, and P10 mice

P2X2 reporter mice were anesthetized with urethane and transcardially perfused at P40, P1, P5, or at P10 with PBS (pH = 7.4), followed by 4% paraformaldehyde (pH = 7.4). Brains were postfixed overnight in 4% paraformaldehyde and sectioned horizontally at 50 µm. The imaging and analyses of all brains were as described earlier for the terminal field labeling experiments.

### PSD95 and CT labeling in the NST

The CT was labeled with crystals of 3 kD tetramethylrhodamine dextran amine at the proximal cut end of the nerve as described previously. Animals were anesthetized 20 h after surgery with urethane and transcardially perfused with PBS (pH = 7.4), followed by 4% paraformaldehyde (pH = 7.4). Brains were postfixed in the same fixative for 4 h and sectioned horizontally at 50 µm. Sections were rinsed and incubated in rabbit anti-PSD95 (1:200, Cat# 51–6900, RRID:AB_2533914; Thermo Fisher Scientific) with 1% BSA, 0.2% Triton X-100, and 0.05% sodium azide in PBS for 48 h at 4°C. Sections were then incubated in donkey anti-rabbit Alexa Fluor 488 (1:400, Cat# 711-545-152, RRID:AB_2313584; Jackson ImmunoResearch Labs) overnight at 4°C. Sections were rinsed after application of  primary and secondary antibodies for six times (15 min/each) with 0.01 M PBS. Sections were then mounted and coverslipped with Vectorshield Hardset mounting medium (Vector Labs).

Sections were imaged with a Nikon 80i Microscope fitted with a Nikon C2 Scanning System (Nikon Instruments) and a 60× oil objective (numerical aperture [NA], 1.40; Plan Apochromat VC, Nikon). CT labels were matched for the wavelengths of the laser in the system (DPSS laser: 561 nm; 10 mW). Sequential optical sections were captured every 0.2 µm for each 50 µm section, and only a range of 5-µm optical sections in the middle of each stack was used for analyses. 3D Deconvolution software (Nikon Elements) was used on each image stack. The procedure to quantify PSD95^+^ labeling and colocalization with CT labels was done with the customized ImageJ software presented in the section Tissue preparation and analysis of NST and LGN terminal field sizes in postnatal mice.

### Neurophysiological taste responses from the CT

Following anesthetization, as described for terminal field labels, the animals were tracheotomized and placed on a circulating water heating pad to maintain body temperature. Hypoglossal nerves were transected bilaterally to prevent tongue movement and the mouse was placed in a nontraumatic head holder. The left CT was isolated using a mandibular approach. The nerve was exposed near the tympanic bulla, cut, desheathed, and positioned on a platinum electrode. A second electrode was placed in a nearby muscle to serve as the ground. Kwik-Sil was placed in the cavity around the nerve.

Whole nerve CT activity was fed to a high impedance input stage amplifier and then led to a PowerLab A/D converter and amplifier and analyzed with PowerLab Scope software (ADInstruments). The output of the PowerLab was fed to an audio monitor and a computer monitor for monitoring activity.

All chemicals were reagent grade and prepared in artificial saliva (Hellekant et al., 1985). Neural responses from the CT were recorded to ascending concentrations series of 0.05, 0.1, 0.25, and 0.5 M NaCl, to 10, 20, and 50 mM citric acid, then to 0.1, 0.25, 0.5 and 1.0 M sucrose, and finally to 10, 20, 50, and 100 mM quinine hydrochloride to assess the taste responses to prototypical stimuli that represent salty, sour, sweet, and bitter, respectively, to humans. Each concentration series was bracketed by applications of 0.5 M NH_4_Cl to monitor the stability of each preparation and for normalizing taste responses. Solutions were applied to the tongue in 5-ml aliquots with a syringe and allowed to remain in the tongue for ∼20 s. We used this period of stimulation so that we could ensure enough time to measure steady-state responses. After each solution application, the tongue was rinsed with artificial saliva for ≥1 min. This period allowed a full recovery of neural responses (i.e., the responses were not adapted by previous responses). In addition, responses were recorded with the NaCl concentration series in the ENaC blocker, amiloride (50 µM). Rinses during this series were with amiloride.

CT responses were calculated as follows: the average voltage of the spontaneous activity that occurred during the second before stimulus onset was subtracted from the voltage that occurred from the period during the first to sixth second after stimulus application. Response magnitudes were then expressed as ratios relative to the mean of 0.5 M NH_4_Cl responses before and after stimulation. Whole nerve response data were retained for analysis only when 0.5 M NH_4_Cl responses that bracketed a concentration series varied by <10%.

### Taste-related behavioral tests

#### Two-bottle preference testing

Two-bottle preference tests between distilled water and a solution of NaCl in distilled water were done in adult control and E3–E12 diet mice. Preferences between two spouts delivering distilled water and a solution of NaCl in distilled water (0.05, 0.075, 0.01, 0.15, 0.3, and 0.45 M) were done in the animal’s home cage. Measures (milliliters consumed) were taken for 48 h followed by a 24-h recovery period where distilled water was available on both spouts. This procedure was followed by successively higher concentrations of NaCl. After a 48-h period of exposure to both bottles containing distilled water, the same procedure was done for sucrose (0.01, 0.03, 0.1, 0.3, and 1.0 M) and then citric acid (1, 3, 10, and 30 mM).

#### Brief access taste-related behavioral response testing

Procedures like that described in detail by Glendinning et al. (2002) were used. Briefly, under 23.5 h water restriction, mice were trained to lick at a sipper tube located just outside the testing chamber (MED-DAV-160M; Davis Rig, Med Associates, Inc.). Access to the tube was provided by opening the shutter for a period of 5 s. Control of the shutter, access to specific stimuli, and counts of licks/presentation were controlled by a computer and software supplied by the manufacturer. Stimuli were presented in randomized blocks, with distilled water presentations delivered for 3 s between each stimulus. Mice were tested for three consecutive days and the average responses (number of licks to a stimulus/average number of licks to distilled water) were analyzed for the last 2 d. This procedure was used to test taste responses to a concentration of NaCl (0.005, 0.01, 0.1, 0.2, 0.6, and 1.0 M) and to quinine hydrochloride (0, 0.006, 0.03, 0.06, 0.1, 0.3, and 1.0 mM). A concentration series of sucrose (0, 0.1, 0.2, 0.3, 0.6, and 1.0 M) was tested similarly, but the denominator used to calculate the response was based on each mouse’s maximal lick rate obtained when they were initially trained to lick at the spout. Procedures for the sodium appetite test were the same as for the NaCl and quinine testing, with the exception that mice received two injections of furosemide (1 h apart; i.p.; 66.7 µg/g body weight, each injection; F4381; Sigma-Aldrich) and fed the low-sodium diet (0.03%) for 48 h before testing. Animals with this test were always given ad libitum access to distilled water. The concentration series of NaCl for this test was (0, 0.05, 0.1, 0.3, 0.5, and 0.7 M). Finally, all animals were given at least 2 d between tests involving different chemicals (e.g., between testing the quinine and sucrose series).

Individual housing of mice was instituted for both sets of experiments to track individual mice and to control for water and/or food restriction (if required) throughout the period of testing. Mice in the two-bottle testing were maintained on food and distilled water ad libitum. Short-access testing involved 23 h water deprivation before each 30-min test, except for sodium appetite. Following short-access testing (e.g., to NaCl), mice were allowed access to tap water and rodent chow for 30 min. Food was made available to all mice ad libitum. Short-access testing occurred on five consecutive weekdays, followed by ad libitum access to water and food on the weekends. Sodium appetite was induced by two injections of furosemide (4381; Sigma-Aldrich; 6.67 μl/injection; i.p.) within 2 h. Mice were then given ad libitum access to distilled water and the low-sodium diet used in the E3–E12 diet mice (i.e., 0.03% NaCl). The same testing schedule then occurred for these mice as described for other short-access tests. Mice on all behavioral tests were weighed daily and were removed from the testing if body weights went below 85% of their pretesting weight. No animal’s body weight reached this level.

### Microglia density analyses and categorization

Heterozygous Cx3Cr1 mice (B6.129P2(Cg)-*Cx3cr1*^*tm1Litt*^/J; #005582; Jackson Labs) were used to determine microglia densities, microglia shapes, and the amount of CD68^+^ label.

Transmitted light images were taken from each section in which microglia were counted and were used to draw the region of interest within the rostral NST. Using Neurolucida (v.5.65; Microbrightfield), an outline of the rostral NST was made along the outer border of the NST and extending 500 µm caudally from the rostral pole to a point and parallel to the major axis of the NST. The open caudal ends of the line were then closed to define the region of interest. For all ages, this region of interest encompassed the terminal fields of all three nerves. See Sun et al. (2015) for descriptions of dorsal and ventral NST zones. An outline of the caudal NST was made similarly, with the exception that there was a reference line drawn 350 µm rostrally, beginning at the caudal-most level of the fourth ventricle. For tissue from E18.5 mice, the same procedure was used with the exception that the lines extended only 200 µm for both rostral and caudal NST measurements. For the LGN, the right LGN was outlined with Neurolucida in sections of interest that contained both ipsilateral and contralateral inputs. Finally, measurements were taken from the dorsal portion of the medulla of E12.5 control and E3–E12 diet mice (200 µm sections) by drawing a 350 µm line parallel to the fourth ventricle and bisecting the medulla. The region of interest was ∼200,000 µm^2^. Transmitted light images were also used to locate and define the outline of the hypoglossal nucleus, which was in or near the ventral NST section. For all counts, the dissector was placed approximately in the middle of the confocal stack (12 µm) guard zone and consisted of two parallel frames spaced 9 µm apart. Image frames were acquired with a 10× objective. Counts of cell soma were done with Neurolucida and the area of the outlined region of interest was used to calculate microglia densities (number of microglia/area of region of interest). Additional animals were used to analyze the types of microglia morphologies and indices of activation. Categorization of microglia was done as previously described (Schafer et al., 2012), which used microglia morphologies and amount of CD68^+^ immunoreactivity.

### P2X2 immunohistochemistry for analyses of P2X2, CD68, IL-33, and IL-34

Mice were transcardially perfused with PBS, followed by 4% paraformaldehyde for 10 min, and postfixed 4 h at 4°C. Please refer to the previous section of Tissue preparation and analysis of NST and LGN terminal field sizes in postnatal mice for a detailed procedure. The antibodies used were goat anti-IL-33 (Cat# PA5-47007, RRID:AB_2606911; Thermo Fisher Scientific), rabbit anti-IL-34 (Cat# PA5-95624, RRID:AB_2807426; Thermo Fisher Scientific), and rat anti-CD68 (Cat# MCA1957, RRID:AB_322219; Bio-Rad, Life Science). P2X2 antibodies raised in guinea pig (Cat# ab10262, RRID:AB_297001; Abcam) or rabbit (Cat# APR-003, RRID:AB_2040054; Alomone Labs) were used to detect gustatory projection to NST indirectly as described previously (Bartel, 2012). Mice were perfused under a surgical microscope with 0.01 M PBS for 2 min followed by 4% paraformaldehyde for 10 min. Brains were postfixed for 2 d at 4°C and sectioned with a Vibratome at 100 μm.

### Microglia in embryonic tissue

Pregnant mice at E9.5, E12.5, and E18.5 were anesthetized, embryos were removed from the uterus, and then fixed in 4% paraformaldehyde for 24 h. Tissue surrounding embryos were removed and embryos were then placed in the fixative for 1 wk. For E9.5 embryos, the whole animal was mounted directly on the slide. For E12.5 and E18.5 embryos, brains were removed from the skull and fixed for 10 d before being sectioned at 200 μm with a vibratome. E9.5 embryos and sections of E12.5 and E18.5 were mounted on slides and coverslipped. E9.5 embryos were imaged with a confocal laser microscope with a 10× objective, and sections from E12.5 and E18.5 were imaged with 10× and 20× objectives.

### Microglia isolation and quantification and statistical analyses for RNA-seq

The rostral half of the left and right NST was dissected from the fresh brains of control and E3–E12 diet mouse litters at P15. NSTs were pooled from six littermates with respect to treatment so that each biological replicate is representative of one litter. In total, three litters were used from control and E3–E12 diet mice. Pooled NSTs were placed in 1 ml HBSS (containing Mg and Ca), 4 U/ml papain, and 50 U/ml DNase-I (#10104159001; Sigma-Aldrich) in a 1.5-ml Eppendorf tube. Samples were then incubated at 37°C for 15 min, followed by gentle trituration five times with a 1,000-μl Eppendorf pipette. Samples were again incubated at 37°C for 15 min, followed by a second gentle trituration. At this point, cells were well dissociated. The 1 ml suspension was then filtered gently through a 70-µm cell strainer and placed in 15-ml tubes containing 14 ml DMEM/F12 with 10% FBS. Cells were then pelleted at 300 relative centrifugal force for 10 min. Cells were then labeled with microglia CD11b^+^ magnetic selection beads (#130-097-142; Miltenyi Biotech). Cells were positively selected by AutoMACS twice using the Possel setting. All 15-ml tubes before use were coated with sterile BSA in diethyl pyrocarbonate–treated PBS to ensure optimal recovery of microglia. After the final positive selection, flow cytometry was used to ensure the purity of the positive and negative fraction of AutoMACS sorted cells. 5% of each fraction was isolated and stained for 15 min at 4°C using antibodies against CD11b (PE-Cy7) CD45 (#BB515; both from R&D Systems), as well as the viability dye ZombieNIR (BioLegend) and Hoechst dye (Cat# H3569, RRID:AB_2651133; Thermo Fisher Scientific) to label all nucleated cells. Cells were then washed and spun at 300 relative centrifugal force for 10 min at 4°C and resuspended in 1% paraformaldehyde in PBS and run on a Gallios flow cytometer (Becton Dickinson). Cells gated on Hoechst positivity, singlet events, and live cells were >90% CD45/CD11b^+^ microglia in the positive fraction with the negative fraction <1% CD45/CD11b^+^.

RNA of positively selected cells was collected using the RNAqueous-Micro Total RNA Isolation kit (#AM1931; Thermo Fisher Scientific) according to the manufacturer’s protocol and stored at −80°C until use. RNA integrity was verified using the Qubit RNA assay kit (Life Technologies) and the Agilent RNA Pico kit (Agilent). Amplification and cDNA construction were performed using Nugen Ovation RNA-seq V2 kit (Tecan) with the manufacturer’s recommended protocol. Sample clean-up after amplification was performed using Qiagen PCR purification kit (Qiagen). Paired-end (50-bp reads) RNA-seq was performed using an Illumina HiSeq (Illumina) yielding 50–70 million reads per sample. All procedures following the acquisition of RNA by the RNAqueous micro kit were performed by HudsonAlpha.

For statistical and graphical analysis of RNA-seq reads, we used the R (v.3.3.2) package DESeq2 of the Bioconductor suite. Adjusted P values <0.1 were used for subsequent downstream analysis. We curated differentially expressed genes using a combination of functional determination (predicted or known) via Uniprot and text/literature mining (Morioka et al., 2018). Statistical validation of functional categories was performed via Fisher’s exact test (Morioka et al., 2018) and independently validated using the “compute overlaps” function of gene set enrichment analysis (GSEA; Table S1).

### Analyses for engulfment experiments

For the engulfment experiment of P22 and P40 with P2X2 and CD68, one section where the hypoglossal nucleus is located was chosen for analysis. A 60× objective (Plan Apo, NA = 1.40) attached to a C2 Nikon laser scanning microscope system was used to collect three stack files (medial, caudal, and lateral to the “core” region of the terminal field) of 7 μm each with a Z-step size of 0.2 μm. Each stack file was 3D deconvoluted and processed using ImageJ and Imaris software (v.10.0; Oxford Instruments). Microglia phagocytosis assays and analyses were done as described previously (Schafer et al., 2012, 2014). 3D volume surface renderings for microglia, P2X2^+^, and CD68^+^ were done with Imaris software and the total volumes of microglia, P2X2^+^, and CD68^+^ were calculated. The percentage of engulfment was calculated in two procedures: (1) the volume of all large microglia (categories 2–5; Fig. 3 E) determined through Imaris was used to normalize the volumes of P2X2^+^ and CD68^+^ to the microglia area, and (2) the volume of the entire region of interest for each section (860,105 µm^3^) was used to normalize the volumes of P2X2^+^ and CD68^+^ labels. These ratios were then multiplied by 100 to yield the percentage of engulfment within microglia. The mean (± SEM) number of large microglia/animal for P22 control, P22 E3–E15 diet, adult control, and adult E3–E15 diet were 22.2 (3.5), 19.5 (3.2), 21.9 (3.2), and 19.0 (0.5), respectively. There were no age- or diet-related differences (P > 0.05). Samples of microglia imaged here were used as examples of microglia categories shown in Fig. S3 A (categories 1–4) because of the high quality of images obtained by imaging with 60× instead of 10× objectives. No example of a category 5 microglia was found in these samples, which is consistent with this category representing <1% of the total microglia categorized.

### RNA isolation and quantitative real-time PCR

Total RNA was extracted using RNeasy mini kit (#74104; Qiagen). Traces of DNA were eliminated in samples by treatment with DNase I. Total RNA was analyzed as described in detail in Sun et al. (2015). Reverse transcription was performed using 2,000 U Superscript III Reverse Transcriptase (#18064022; Thermo Fisher Scientific) and 50 ng random hexamers in 25 ml reaction volumes following the manufacturer’s protocol with the same amount (50 ng) of total RNA. qPCR was performed by 7500 Fast Real-Time PCR System (Thermo Fisher Scientific) using the Taq-Man Universal PCR Kit. Assays of C1q (#4331182, C1qa, Assay ID: Mm00432142_m1) and mouse glyceraldehyde 3-phosphate dehydrogenase (GAPDH; # 4331182, Assay ID: Mm99999915_g1) were purchased from Thermo Fisher Scientific. PCR efficiencies were determined by performing PCR with serial (10-fold) dilutions of cDNA in parallel. All samples were run in parallel with the housekeeping gene GAPDH to normalize cDNA loading. Each assay was carried out in triplicate. PCR was performed for 40 cycles at 95°C for 15 s and at 60°C for 1 min. The comparative 2^–ΔΔCT^ method was used to determine the relative gene expression levels of the A, B, and C chains of *C1q* (Sun et al., 2015).

### C1q immunohistochemistry in P15, P25 mice, and P40

An antibody to C1q (rabbit; 1:1,000; Cat# ab182451, RRID:AB_2732849; Abcam) was used to examine the overlap of staining in P25 mice. Standard perfusion and fixation procedures were followed and described above. Elements software (v.5.02; Nikon) was used to automatically draw a region of interest around the densest P2X2^+^ label (gustatory projection) and draw a region of interest around all the P2X2^+^ label. These two regions of interest were then used to calculate the density of C1q^+^ label within each of the two regions of interest. From these measures, the densities within the entire P2X2^+^ field, within the core of the P2X2^+^ field, and of the C1q^+^ label between the core and outermost region of interest (i.e., around the fringe of the terminal field) were determined.

### Electron microscopy

Four C57BL/6J P18 mice were anesthetized with urethane and perfused transcardially with Tyrode’s solution (137 mm NaCl, 2 mm KCl, 0.9 mm CaCl_2_, 1.2 mm MgCl_2_, 11.9 mm NaHCO_3_, 0.4 mm NaH_2_PO_4_, 5.5 mm glucose, 281 mOsm, pH 7.4) via the left ventricle for 1–2 min. This was followed by fixation with 4% freshly depolymerized paraformaldehyde and 0.05% glutaraldehyde in 0.1 M phosphate buffer (PB) for 10 min. Brains were removed from skulls and postfixed in 4% paraformaldehyde for 2 h at 4°C, blocked, and then sectioned with a vibratome at a thickness of 50 μm.

Washed sections were placed in PB containing 0.1% sodium borohydride (NaBH_4_) for 15 min to inactivate residual aldehyde. Sections were then washed with PB at least 4× until the solution was clear of bubbles and then followed by 0.05% Triton X-100 in PB for 30 min. Sections were incubated in Aurion blocking solution (#25596; Electron Microscopy Science) containing 5% normal goat serum, 5% BSA, and 0.1% cold water fish skin gelatin for 1 h and then rinsed with PBS/BSA-c (0.2% BSA-c in 0.01 M PBS), 2 × 10 min. After blocking, sections were incubated in a mixture of primary antibodies of rabbit anti-C1q (1:1,000, #ab182451; Abcam) and guinea pig anti-p2x2 (1:500, #ab10262; Abcam) in PBS/BSA-c with 0.05% NaN_3_ at 4°C for 72 h. After rinsing several times, sections were incubated in goat anti-GP biotinylated (1:200, Cat#PK-4007, RRID:AB_2336816; Vector Laboratories) with F(ab′)2 goat anti-rabbit IgG UltraSmall gold (1:100, # 25361; EMS) in PBS/BSA-c with 0.05% NaN_3_ overnight. Sections were incubated in ABC (Vector Laboratories) complex for 1 h at RT and then rinsed with PBS. After 10 min, sections were rinsed with PB followed by condition solution (#25830; ECS, EMS) for 4 × 10 min. Silver enhancement (Cat. 25521; R-GENT SE-EM) was performed according to the manufacturer’s recommendation for 90 min. Silver enhancement was stopped by transferring sections to 0.03 M sodium thiosulfate in ECS for 10 min followed by ECS and PB rinse. Sections were postfixed in 2.5% glutaraldehyde in PB for 2 h and rinsed by PB. All immuno-incubations were done on a gentle shaker at 4°C and rinsed with PBS/BSA-c six times × 10 min. Sections were fixed with 0.5% osmium tetroxide for 15 min, dehydrated, and flat-embedded in Epon resin between two sheets of Aclar film (EMS). After resin polymerization, small pieces of flat-embedded sections containing the rostral NST were dissected and mounted on plastic capsules. Ultrathin sections at the interface of tissue and Epon were collected and counterstained with filtered 2% lead citrate for contrast. Ultrathin sections were examined on a JEOL 1010 electron microscope. A 16 M pixel digital camera (SIA-12C, Scientific Instruments and Applications) was used to capture images at 12,000× magnification.

### Quantification and statistical analyses

Apart from RNA-seq reads, statistical analyses of all measurements were done with multiple unpaired *t* tests corrected for multiple comparisons using the Holm–Sidak method (P < 0.05; Prism 6 for Mac OSX; Graphpad Software).

### Online supplemental material

We are including five supplemental figures and five tables that complement and extend the findings and figures contained in the manuscript. Fig. S1 shows expanded photomicrographs of terminal fields in the NST, validation of P2X2^+^ as a proxy for CT and GSP labels, animal, and gustatory structural indices, and dLGN measurements. Fig. S2 shows neurophysiological taste responses and behavioral measures where controls and E3–E12 diet mice were similar. Fig. S3 shows microglia activity categorization, densities in unaffected brain regions, isolation of microglia, and measures of engulfment of P2X2^+^ terminals and CD68^+^. Fig. S4 shows expanded photomicrographs of terminal fields in the NST in control mice treated with PLX5622 or E3–E12 diet mice treated with m-csf. Fig. S5 demonstrates that IL-33 and IL-34 also show age- and diet-related differences in the rostral NST. Tables S1, S2, and S3 elaborate on the data and method used for the RNA-seq findings. Tables S4 and S5 are tables of extensive statistical analyses of C3 and C1q knockout mice and C1q immunohistochemical experiments.

## Supplementary Material

Table S1 shows GSEA upregulated in E3–E12 sodium-restricted mice.

Table S2 shows genes upregulated in E3–E12 sodium-restricted mice.

Table S3 shows downregulated in E3–E12 sodium-restricted mice.

Table S4 shows statistical comparisons of C1q and C3 knockout mice with controls and E3–E12 diet mice mean (±SEM).

Table S5 shows immunohistochemical statistical comparisons for C1q^+^ label.

## Data Availability

Requests for further data beyond that provided in the paper and additional information regarding this study should be directed to and will be fulfilled by the corresponding author. The RNA-seq data generated during this study are available at Gene Expression Omnibus accession no. GSE239942.
