# Supplementary material for: Maternal diet during early gestation influences postnatal taste activity–dependent pruning by microglia
Source: J Exp Med. 2023 Sep 21;220(12):e20212476. doi: 10.1084/jem.20212476 (PMC10512853; doi:10.1084/jem.20212476)
Supplement: Table S1 — shows GSEA upregulated in E3–E12 sodium-restricted mice. [file JEM_20212476_TableS1.pdf]

Table S1-- Gene Set Enrichment Analysis: Upregulated In E3-E12 Sodium-Restricted Mice

Gene Set Name	# Genes in Gene Set (K)	# Genes in Overlap (k)	k/K	p-value	FDR q-value
GOBP REGULATION OF TRANSPORT	1793	19	0.011	4.77E-10	4.05E-06
GOBP IMMUNE SYSTEM DEVELOPMENT	1029	15	0.015	6.06E-10	4.05E-06
GOBP REGULATION OF RESPONSE TO STRESS	1437	17	0.012	8.90E-10	4.05E-06
GOBP POSITIVE REGULATION OF PROTEIN METABOLIC PROCESS	1520	17	0.011	2.07E-09	7.07E-06
GOBP REGULATION OF CELL POPULATION PROLIFERATION	1779	18	0.010	3.08E-09	8.00E-06
GOBP CELL MOTILITY	1794	18	0.010	3.52E-09	8.00E-06
REACTOME PLATELET ADHESION TO EXPOSED COLLAGEN	15	4	0.267	1.46E-08	2.85E-05
GOBP REGULATION OF CELLULAR LOCALIZATION	954	13	0.014	2.13E-08	3.01E-05
GOBP POSITIVE REGULATION OF MULTICELLULAR ORGANISMAL PROCESS	1556	16	0.010	2.15E-08	3.01E-05
GOCC CELL SURFACE	957	13	0.014	2.20E-08	3.01E-05
GOBP REGULATION OF PROTEIN MODIFICATION PROCESS	1584	16	0.010	2.76E-08	3.42E-05
GOBP POSITIVE REGULATION OF GENE EXPRESSION	1176	14	0.012	3.09E-08	3.51E-05
GOBP REGULATION OF PHOSPHORUS METABOLIC PROCESS	1427	15	0.011	4.77E-08	4.58E-05
GOBP REGULATION OF ESTABLISHMENT OF PROTEIN LOCALIZATION	525	10	0.019	4.86E-08	4.58E-05
GOCC SIDE OF MEMBRANE	678	11	0.016	5.03E-08	4.58E-05
GOBP APOPTOTIC PROCESS	1933	17	0.009	7.03E-08	6.00E-05
GOBP CELLULAR RESPONSE TO STRESS	1978	17	0.009	9.78E-08	7.81E-05
GOBP POSITIVE REGULATION OF TRANSPORT	903	12	0.013	1.03E-07	7.81E-05
GOBP REGULATION OF INTRACELLULAR SIGNAL TRANSDUCTION	1767	16	0.009	1.23E-07	8.85E-05
WP NUCLEAR RECEPTORS METAPATHWAY	314	8	0.026	1.30E-07	8.85E-05
GOCC INTRINSIC COMPONENT OF PLASMA MEMBRANE	1795	16	0.009	1.52E-07	9.91E-05
GOCC EXTERNAL SIDE OF PLASMA MEMBRANE	455	9	0.020	1.74E-07	1.08E-04
GOBP POSITIVE REGULATION OF CELL POPULATION PROLIFERATION	986	12	0.012	2.63E-07	1.56E-04
GOBP GENERATION OF PRECURSOR METABOLITES AND ENERGY	505	9	0.018	4.16E-07	2.37E-04
GOBP REGULATION OF MULTICELLULAR ORGANISMAL DEVELOPMENT	1471	14	0.010	4.70E-07	2.53E-04
GOBP ORGANIC ACID TRANSMEMBRANE TRANSPORT	156	6	0.039	4.82E-07	2.53E-04

GOBP POSITIVE REGULATION OF SIGNALING	1758	15	0.009	6.88E-07	3.35E-04
GOBP REGULATION OF IMMUNE SYSTEM PROCESS	1523	14	0.009	7.11E-07	3.35E-04
BIOCARTA FREE PATHWAY	10	3	0.300	7.27E-07	3.35E-04
GOBP VASCULAR PROCESS IN CIRCULATORY SYSTEM	270	7	0.026	7.36E-07	3.35E-04
GOBP REGULATION OF PROTEIN LOCALIZATION TO MEMBRANE	172	6	0.035	8.54E-07	3.77E-04
GOBP PROTEOLYSIS	1795	15	0.008	8.92E-07	3.81E-04
GOBP REGULATION OF CELLULAR RESPONSE TO STRESS	729	10	0.014	9.83E-07	3.98E-04
GOBP POSITIVE REGULATION OF PHOSPHORUS METABOLIC PROCESS	916	11	0.012	9.92E-07	3.98E-04
GOBP POSITIVE REGULATION OF MOLECULAR FUNCTION	1591	14	0.009	1.19E-06	4.65E-04
GOBP RESPONSE TO ABIOTIC STIMULUS	1149	12	0.010	1.31E-06	4.99E-04
GOBP REGULATION OF PROTEIN LOCALIZATION TO PLASMA MEMBRANE	103	5	0.049	1.45E-06	5.36E-04
GOBP NEGATIVE REGULATION OF MOLECULAR FUNCTION	1174	12	0.010	1.64E-06	5.90E-04
GOBP CIRCULATORY SYSTEM PROCESS	610	9	0.015	1.97E-06	6.64E-04
GOBP POSITIVE REGULATION OF CATALYTIC ACTIVITY	1196	12	0.010	1.99E-06	6.64E-04
KEGG FOCAL ADHESION	199	6	0.030	1.99E-06	6.64E-04
GOBP ESTABLISHMENT OF PROTEIN LOCALIZATION	1668	14	0.008	2.07E-06	6.73E-04
GOBP REGULATION OF CELL DEATH	1689	14	0.008	2.39E-06	7.61E-04
GOBP POSITIVE REGULATION OF INTRACELLULAR SIGNAL TRANSDUCTION	1014	11	0.011	2.64E-06	8.12E-04
GOBP NEGATIVE REGULATION OF RESPONSE TO STIMULUS	1707	14	0.008	2.71E-06	8.12E-04
GOBP POSITIVE REGULATION OF TRANSCRIPTION BY RNA POLYMERASE II	1235	12	0.010	2.77E-06	8.12E-04
GOBP POSITIVE REGULATION OF PROTEIN MODIFICATION PROCESS	1020	11	0.011	2.80E-06	8.12E-04
WP HIPPOMERLIN SIGNALING DYSREGULATION	121	5	0.041	3.21E-06	9.15E-04
GOBP REGULATION OF PROTEIN LOCALIZATION TO CELL PERIPHERY	125	5	0.040	3.77E-06	1.05E-03
GOBP AMEBOIDAL TYPE CELL MIGRATION	495	8	0.016	3.91E-06	1.07E-03
GOBP CENTRAL NERVOUS SYSTEM DEVELOPMENT	1061	11	0.010	4.07E-06	1.09E-03
GOBP RESPONSE TO EXTRACELLULAR STIMULUS	505	8	0.016	4.53E-06	1.18E-03
GOBP REGULATION OF PHOSPHOPROTEIN PHOSPHATASE ACTIVITY	59	4	0.068	4.59E-06	1.18E-03

GOMF PROTEIN CONTAINING COMPLEX BINDING	1302	12	0.009	4.74E-06	1.20E-03
GOBP REGULATION OF CELL DEVELOPMENT	516	8	0.016	5.30E-06	1.32E-03
REACTOME OTHER SEMAPHORIN INTERACTIONS	19	3	0.158	5.80E-06	1.41E-03
GOMF PROTEASE BINDING	139	5	0.036	6.33E-06	1.52E-03
GOBP FAT CELL DIFFERENTIATION	245	6	0.025	6.59E-06	1.55E-03
GOBP NEGATIVE REGULATION OF CELL DIFFERENTIATION	719	9	0.013	7.44E-06	1.72E-03
KEGG ADIPOCYTOKINE SIGNALING PATHWAY	67	4	0.060	7.64E-06	1.74E-03
GOBP TELENCEPHALON DEVELOPMENT	257	6	0.023	8.65E-06	1.92E-03
GOBP CELL ACTIVATION	1150	11	0.010	8.69E-06	1.92E-03
GOMF CELL ADHESION MOLECULE BINDING	559	8	0.014	9.47E-06	2.05E-03
GOBP MITOTIC CELL CYCLE	950	10	0.011	1.02E-05	2.17E-03
GOBP NEGATIVE REGULATION OF NEURON DIFFERENTIATION	73	4	0.055	1.08E-05	2.26E-03
GOBP REGULATION OF INFLAMMATORY RESPONSE	417	7	0.017	1.28E-05	2.64E-03
PID LYMPH ANGIOGENESIS PATHWAY	25	3	0.120	1.37E-05	2.78E-03
GOBP HOMEOSTATIC PROCESS	1708	13	0.008	1.43E-05	2.87E-03
GOBP RESPONSE TO XENOBIOTIC STIMULUS	432	7	0.016	1.60E-05	3.17E-03
GOBP NEGATIVE REGULATION OF CATALYTIC ACTIVITY	796	9	0.011	1.67E-05	3.25E-03
GOBP ASTROCYTE DIFFERENTIATION	82	4	0.049	1.71E-05	3.28E-03
KEGG HYPERTROPHIC CARDIOMYOPATHY HCM	83	4	0.048	1.79E-05	3.40E-03
GOBP REGULATION OF RESPONSE TO EXTERNAL STIMULUS	1017	10	0.010	1.83E-05	3.42E-03
GOBP REGULATION OF PHOSPHATASE ACTIVITY	84	4	0.048	1.88E-05	3.42E-03
GOBP WOUND HEALING	444	7	0.016	1.91E-05	3.42E-03
GOBP POSITIVE REGULATION OF PROTEIN MATURATION	28	3	0.107	1.94E-05	3.42E-03
GOBP REGULATION OF ANION TRANSMEMBRANE TRANSPORT	28	3	0.107	1.94E-05	3.42E-03
GOBP REGULATION OF HYDROLASE ACTIVITY	1025	10	0.010	1.95E-05	3.42E-03
GOBP HEAD DEVELOPMENT	816	9	0.011	2.03E-05	3.43E-03
GOBP NEGATIVE REGULATION OF CELL DEATH	1031	10	0.010	2.05E-05	3.43E-03
WP CLEAR CELL RENAL CELL CARCINOMA PATHWAYS	86	4	0.047	2.06E-05	3.43E-03
GOBP POSITIVE REGULATION OF PROTEIN LOCALIZATION	450	7	0.016	2.08E-05	3.43E-03
GOBP VASCULATURE DEVELOPMENT	819	9	0.011	2.09E-05	3.43E-03

GOBP REGULATION OF DNA BINDING TRANSCRIPTION FACTOR ACTIVITY	454	7	0.015	2.20E-05	3.58E-03
GOBP POSITIVE REGULATION OF CELL DEVELOPMENT	307	6	0.020	2.36E-05	3.79E-03
GOBP POSITIVE REGULATION OF ESTABLISHMENT OF PROTEIN LOCALIZATION	309	6	0.019	2.45E-05	3.87E-03
KEGG DILATED CARDIOMYOPATHY	90	4	0.044	2.46E-05	3.87E-03
GOBP GLIOGENESIS	310	6	0.019	2.49E-05	3.87E-03
GOMF XENOBIOTIC TRANSMEMBRANE TRANSPORTER ACTIVITY	31	3	0.097	2.65E-05	4.06E-03
GOBP NEGATIVE REGULATION OF NUCLEOBASE CONTAINING COMPOUND METABOLIC PROCESS	1551	12	0.008	2.71E-05	4.11E-03
GOMF PROTEIN DIMERIZATION ACTIVITY	1067	10	0.009	2.75E-05	4.12E-03
GOBP INFLAMMATORY RESPONSE	850	9	0.011	2.79E-05	4.12E-03
GOBP REGULATION OF ANION TRANSPORT	93	4	0.043	2.80E-05	4.12E-03
GOBP REGULATION OF ASTROCYTE DIFFERENTIATION	32	3	0.094	2.92E-05	4.21E-03
GOBP REGULATION OF PROTEIN DEPHOSPHORYLATION	94	4	0.043	2.93E-05	4.21E-03
GOBP RESPONSE TO HORMONE	863	9	0.010	3.14E-05	4.46E-03
WP OXIDATIVE STRESS RESPONSE	33	3	0.091	3.21E-05	4.51E-03
GOBP ORGANIC ACID TRANSPORT	326	6	0.018	3.30E-05	4.60E-03
GOMF COLLAGEN BINDING INVOLVED IN CELL MATRIX ADHESION	5	2	0.400	3.38E-05	4.63E-03
GOCC ANCHORING JUNCTION	1333	11	0.008	3.39E-05	4.63E-03

**Gene Set Enrichment Analysis: Downregulated In E3-E12 Sodium-Restricted Mice**					
GOMF CELL ADHESION MOLECULE BINDING	559	9	0.016	6.53E-08	8.92E-04
GOMF CADHERIN BINDING	334	7	0.021	3.67E-07	2.51E-03
GOCC INTRINSIC COMPONENT OF ENDOPLASMIC RETICULUM MEMBRANE	172	5	0.029	3.93E-06	1.79E-02
