# Supplementary material for: Maternal diet during early gestation influences postnatal taste activity–dependent pruning by microglia
Source: J Exp Med. 2023 Sep 21;220(12):e20212476. doi: 10.1084/jem.20212476 (PMC10512853; doi:10.1084/jem.20212476)
Supplement: Table S2 — shows genes upregulated in E3–E12 sodium-restricted mice. [file JEM_20212476_TableS2.pdf]

Table 2 – Genes Upregulated In E3-E12 Sodium-Restricted Mice

Gene	Function	Gene Name	Log 2Fold Change	Adjust. Prob.
Shroom1	Actin/Cytoskeleton	shroom family member 1	1.706	0.040
Mob2	Actin/Cytoskeleton	MOB kinase activator 2	2.055	0.051
Cap1	Actin/Cytoskeleton; Cell Migration; Cell Morphogenesis; Receptor-Mediated Endocytosis	CAP, adenylate cyclase-associated protein 1 (yeast)	1.090	0.070
Fam107a	Actin/Cytoskeleton; Cell Migration; Stress Response	family with sequence similarity 107, member A	1.656	0.012
Ppp1r12a	Cell Adhesion	protein phosphatase 1, regulatory (inhibitor) subunit 12A	1.524	0.041
Cntn2	Cell Adhesion; Actin/Cytoskeleton	contactin 2	1.968	0.039
Vwf	Cell Adhesion; ECM organization; Wound Healing	Von Willebrand factor homolog	1.901	0.000
Hes1	Cell Adhesion; Cell Migration; Differentiation; Morphogenesis	hairy and enhancer of split 1 (Drosophila)	1.626	0.097
Itgb1	Cell Adhesion; Cell Migration; ECM Organization	integrin beta 1 (fibronectin receptor beta)	1.268	0.020
Itga1	Cell Adhesion; Cell Migration; ECM Organization	integrin alpha 1	1.555	0.020
Kank2	Anti-Apoptotic; Actin/Cytoskeleton; Cell Adhesion; Cell Migration; Vitamin D Signaling	KN motif and ankyrin repeat domains 2	1.519	0.043
Cntfr	Anti-Apoptotic; Proliferation; Cell Migration; Microglia Activation	ciliary neurotrophic factor receptor	1.890	0.018
Ly6c1	Cell Migration; Circulating Monocyte-Derived Microglia	lymphocyte antigen 6 complex, locus C1	1.772	0.009
Flt1	Cell Migration; Differentiation; ERK1/2 Signaling; VEGF Signaling	FMS-like tyrosine kinase 1	1.089	0.070
Tmem218	Ciliogenesis; Cell Migration	transmembrane protein 218	1.475	0.076
Rfx3	Ciliogenesis; Cell Migration; Transcription Regulation	regulatory factor X, 3 (influences HLA class II expression)	1.642	0.007
Cd248	Angiogenesis; Cell Proliferation; Cell Migration; PDGFR Signaling	CD248 antigen, endosialin	1.861	0.032
Ptprb	Angiogenesis	protein tyrosine phosphatase, receptor type, B	1.042	0.097

Flt4	VEGF Receptor; Anti-Apoptotic; Angiogenesis; PI3K/AKT Signaling; MAPK/ERK Signaling	FMS-like tyrosine kinase 4	1.730	0.014
Mxd3	Anti-Apoptotic; Proliferation; Transcription Regulation	Max dimerization protein 3	2.163	0.025
Crip2	Proliferation; Differentiation	cysteine rich protein 2	1.656	0.048
Klf13	Anti-Proliferation; Transcription Regulation	Kruppel-like factor 13	1.303	0.014
Bcl9	Canonical Wnt Signaling; Proliferation; Differentiation; Cell Morphogenesis	B cell CLL/lymphoma 9	1.117	0.069
Gli3	Cell Differentiation; Wound Healing; Anti-Apoptotic; Anti-Proliferation	GLI-Kruppel family member GLI3	1.946	0.010
Ttn	Mechanical Force; Mechanosensor; Cell Cycle	titin	2.075	0.002
Slc6a11	GABA Uptake; Beta-Alanine / Taurine Uptake	solute carrier family 6 (neurotransmitter transporter, GABA), member 11	1.231	0.050
Slc38a3	Amino Acid Metabolism	solute carrier family 38, member 3	1.505	0.087
Ugt1a7c	Estrogen Response; Glucuronidation	UDP glucuronosyltransferase 1 family, polypeptide A7C	1.847	0.042
Nr1d1	Circadian Rhythm; Cholesterol Homeostasis; Hormone Signaling; Bile Acid Metabolism; Lipid Metabolism	nuclear receptor subfamily 1, group D, member 1	1.410	0.020
Ptdss1	Phosphatidylserine Synthesis; Lipid Metabolism; Phospholipid Metabolism	phosphatidylserine synthase 1	1.889	0.007
Gramd1a	Lipid Transport; Lipid Metabolism; Cholesterol Transport; Cholesterol Homeostasis	GRAM domain containing 1A	1.853	0.011
Nucb1	Calcium Homeostasis; IGF Transport/Uptake; Insulin Signaling	nucleobindin 1	1.315	0.043
Abcb1a	Carbohydrate Transport; Hormone Transport; Osmoregulation; Anti-Apoptotic	ATP-binding cassette, sub-family B (MDR/TAP), member 1A	2.071	0.000
Adgrf5	Anti-Inflammatory; Phospholipid Synthesis; Glucose Homeostasis	adhesion G protein-coupled receptor F5	1.374	0.013
Gcgr	Glucose Homeostasis; Nutrient Deprivation; Glucagon Signaling; cAMP	glucagon receptor	1.933	0.089

Rsc1a1	Exocytosis; Glucose Transport; Glycolysis	regulatory solute carrier protein, family 1, member 1	2.054	0.051
Aldoc	Glycolysis	aldolase C, fructose-bisphosphate	1.831	0.013
Slc2a1	Glycolysis; Osmoregulation	solute carrier family 2 (facilitated glucose transporter), member 1	1.321	0.008
Eno1	Glycolysis; Oxidative Stress	enolase 1, alpha non-neuron	3.952	0.000
Mtfr1	OXPHOS; Mitochondrial Dynamics	mitochondrial fission regulator 1	1.644	0.063
Nd4l	OXPHOS; Mitochondrial Electron Transport; Complex I	NADH dehydrogenase subunit 4L	1.610	0.008
ND1	OXPHOS; Mitochondrial Electron Transport; Complex I	NADH dehydrogenase subunit 1	1.332	0.019
Suclg2	TCA Cycle; Succinate Metabolism; Succinyl-CoA Metabolism	succinate-Coenzyme A ligase, GDP-forming, beta subunit	1.651	0.061
Nmt1	Ketone Metabolism; Myristoylation	N-myristoyltransferase 1	1.082	0.076
Aldh1a1	Retinal Metabolism; Retinoid Metabolism; Aldehyde Metabolism; Cofactor Metabolism	aldehyde dehydrogenase family 1, subfamily A1	1.872	0.083
Gsr	Oxidative Stress; Glutathione Metabolism	glutathione reductase	2.184	0.005
Gpx1	Oxidative Stress; Glutathione Metabolism; Anti-Apoptotic; Anti-Inflammatory; Angiogenesis; Wound Healing	glutathione peroxidase 1	1.228	0.085
Fcer1g	Antigen Presentation; Fc-Receptor Signaling; Pro-Inflammatory; Antibody-Mediated Phagocytosis	Fc receptor, IgE, high affinity I, gamma polypeptide	1.360	0.004
Nfkbia	Pro-Inflammatory; Anti-Apoptotic; IL-1 Signaling; TNFalpha Signaling; NFkB Signaling	nuclear factor of kappa light polypeptide gene enhancer in B cells inhibitor, alpha	1.053	0.077
Nfkbie	NFkB Signaling; Anti-Inflammatory	nuclear factor of kappa light polypeptide gene enhancer in B cells inhibitor, epsilon	1.228	0.087
Sema7a	Pro-Inflammatory; Cell Migration; Cell Adhesion; Neurogenesis	sema domain, immunoglobulin domain (Ig), and GPI membrane anchor, (semaphorin) 7A	1.754	0.009
Nlrc4	Pro-Inflammatory; IL-1 Signaling; TNFalpha Signaling; NFkB Signaling; Inflammasome; Pyroptosis	NLR family, CARD domain containing 4	1.651	0.081

Tnf	Pro-Inflammatory; Pro-Apoptotic; TNFalpha Signaling	tumor necrosis factor	1.584	0.006
Rack1	TNF Signaling; IGFR Signaling; Cell Cycle; ER Stress/UPR; Wnt Signaling; Pro-Apoptotic; Cell Migration; Ubiquitination; Circadian Rhythm	receptor for activated C kinase 1	1.099	0.096
Dhx9	Pro-Inflammatory; Transcription Regulation; Translational Regulation	DEAH (Asp-Glu-Ala-His) box polypeptide 9	1.643	0.009
Pabpc4	mRNA Stability	poly(A) binding protein, cytoplasmic 4	1.285	0.089
Edem1	ER Stress/UPR; IRE1; ERAD; Glycoprotein Breakdown	ER degradation enhancer, mannosidase alpha-like 1	2.292	0.000
Kdelr1	Vesicular Transport; ER Stress/UPR; Anti-Apoptotic	KDEL (Lys-Asp-Glu-Leu) endoplasmic reticulum protein retention receptor 1	1.558	0.018
Bod1l	DNA Damage	biorientation of chromosomes in cell division 1-like	2.872	0.000
Gadd45b	DNA Damage; Pro-Apoptotic; Anti-Apoptotic; Cell Cycle	growth arrest and DNA-damage-inducible 45 beta	2.212	0.003
Usp47	DNA Damage; Anti-Apoptotic; Ubiquitination	ubiquitin specific peptidase 47	1.543	0.039
Trip12	DNA Damage; Ubiquitination	thyroid hormone receptor interactor 12	1.586	0.013
Hspbp1	Ubiquitination; Protein Folding; Inhibits HSPA1A	HSPA (heat shock 70kDa) binding protein, cytoplasmic cochaperone 1	1.846	0.080
Fam63a	Ubiquitination	family with sequence similarity 63, member A	1.720	0.008
Klhl18	Ubiquitination	kelch-like 18	2.015	0.018
Commd8	Ubiquitination	COMM domain containing 8	1.701	0.036
Mid1	Ubiquitination	midline 1	1.037	0.099
Ypel5	Ubiquitination; Proliferation	yippee-like 5 (Drosophila)	1.317	0.078
Psmd8	Proteosome; Ubiquitination	proteasome (prosome, macropain) 26S subunit, non-ATPase, 8	1.695	0.053
Vmn2r42	Vomeronasal GPCR	vomeronasal 2, receptor 42	2.365	0.010
H2-Q10	Antigen Presentation	histocompatibility 2, Q region locus 10	1.159	0.039
Rpusd4	Mitochondrial Protein Synthesis	RNA pseudouridylate synthase domain containing 4	1.816	0.077
Dopey1	Vesicular Transport	dopey family member 1	1.497	0.073
Grasp	Recycling Endosomes	GRP1 (general receptor for phosphoinositides 1)-associated scaffold protein	1.458	0.046

Taf1d	Transcriptional Regulation	TATA-box binding protein associated factor, RNA polymerase I, D	2.527	0.005
Hlf	Circadian Rhythm; Transcription Regulation	hepatic leukemia factor	1.640	0.029
L3mbtl2	Chromatin Regulator	l(3)mbt-like 2 (Drosophila)	1.516	0.077
Usp53	Pro-Apoptotic; Tight Junctions	ubiquitin specific peptidase 53	1.383	0.081
