# Supplementary material for: Maternal diet during early gestation influences postnatal taste activity–dependent pruning by microglia
Source: J Exp Med. 2023 Sep 21;220(12):e20212476. doi: 10.1084/jem.20212476 (PMC10512853; doi:10.1084/jem.20212476)
Supplement: Table S3 — shows downregulated in E3–E12 sodium-restricted mice. [file JEM_20212476_TableS3.pdf]

Table S3 -- Downregulated In E3-E12 Sodium-Restricted Mice

Dock3	Actin/Cytoskeleton; Cell Adhesion; Cell Migration	dedicator of cyto-kinesis 3	-1.319	0.083
Notch1	Angiogenesis; Morphogenesis; Cell Migration; ECM; Cell Adhesion	notch 1	-1.033	0.099
Adgrb1	Apoptotic Cell Clearance; Cell Adhesion; Anti-Inflammatory; Angiogenesis	adhesion G protein-coupled receptor B1	-2.068	0.047
Lrrc4c	Cell Adhesion	leucine rich repeat containing 4C	-1.794	0.039
Pnn	Cell Adhesion	pinin	-1.391	0.007
Cldn2	Cell Adhesion; Osmoregulation	claudin 2	-2.183	0.011
Skap2	Cell Adhesion; Proliferation	src family associated phosphoprotein 2	-1.951	0.083
Add1	Actin/Cytoskeleton; Cell Volume Regulation; ER Stress/UPR	adducin 1 (alpha)	-1.064	0.051
Zmym3	Actin/Cytoskeleton; Cell Morphology; DNA Damage	zinc finger, MYM-type 3	-1.427	0.010
Rnd3	Actin/Cytoskeleton; Cell Adhesion; Cell Migration; Cell Shape	Rho family GTPase 3	-1.642	0.048
Kit	Actin/Cytoskeleton; Cell Migration; Pro-Inflammatory; Anti-Apoptotic; Cell Shape; Glycosphingolipid Metabolism; Bile Acid Metabolism; PI3K/AKT Signaling	kit oncogene	-2.180	0.013
Mark2	Cell Polarity; Autophagy; Cytoskeleton; Cell Shape; Cell Migration	MAP/microtubule affinity regulating kinase 2	-2.351	0.000
Palm2	Cell Shape	paralemmin 2	-1.718	0.036
Scai	Cell Migration; Transcription Regulation	suppressor of cancer cell invasion	-1.366	0.097
Kdm4c	Amino Acid Metabolism; Chromatin Regulation; Transcription Regulation	lysine (K)-specific demethylase 4C	-1.816	0.010
Gpaa1	Glycolipid Synthesis; GPI-anchor Synthesis	GPI anchor attachment protein 1	-1.415	0.079
Arglu1	Glucocorticoid Signaling; Stress Hormone Signaling	arginine and glutamate rich 1	-3.407	0.000
Ktn1	Cell Adhesion; IGF Transport/Uptake; Vesicular Transport	kinectin 1	-2.357	0.014
Igfbp2	Hormone Signaling; Estrogen Response; IGF	insulin-like growth factor binding protein 2	-1.849	0.014

	Transport/Uptake; Proliferation; Neuroprotection; Neuronal Stress			
Igfbp5	Hormone Signaling; Estrogen Response; IGF Transport/Uptake; Proliferation; Neuroprotection; Neuronal Stress	insulin-like growth factor binding protein 5	-1.603	0.031
Arfgef3	Hormone Signaling; Estrogen Response; Insulin Signaling	ARFGEF family member 3	-2.034	0.032
Lipo1	Lipid Metabolism	lipase, member O1	-1.400	0.058
Etnk2	Phosphatidylethanolamine Synthesis; Lipid Metabolism; Phospholipid Metabolism	ethanolamine kinase 2	-2.411	0.000
Elovl6	Fatty Acid Metabolism, Lipid Metabolism; Sphingolipid Metabolism	ELOVL family member 6, elongation of long chain fatty acids (yeast)	-1.711	0.032
Tecr	Lipid Metabolism; Fatty Acid Metabolism; Long-Chain Fatty Acid Synthesis	trans-2,3-enoyl-CoA reductase	-1.958	0.000
Fasn	Lipid Metabolism; Fatty Acid Metabolism; Long-Chain Fatty Acid Synthesis	fatty acid synthase	-1.935	0.003
Lonp2	Peroxisome Function; Fatty Acid Oxidation	lon peptidase 2, peroxisomal	-1.215	0.058
Cox16	OXPHOS; Mitochondrial Electron Transport; Complex IV	cytochrome c oxidase assembly protein 16	-1.507	0.012
Slc25a36	OXPHOS; Mitochondrial Membrae Potential; Mitochondrial Genome Maintenance; Pyrimidine Nucleotide Transport	solute carrier family 25, member 36	-1.100	0.099
Ttr	ECM Organization; Retinol/Retinoid Metab; Thyroid Hormone Transport	transthyretin	-2.832	0.000
Papss1	Sulfur Metabolism; Sulfate Assimilation	3'-phosphoadenosine 5'-phosphosulfate synthase 1	-1.986	0.045
Zfp36	mRNA Decay; Anti-Inflammatory; Negative Regulator of TNF; Wound Healing	zinc finger protein 36	-1.243	0.076
Sppl2b	Pro-Inflammatory; Anti-Inflammatory; TNFalpha Regulation	signal peptide peptidase like 2B	-2.113	0.004
Hmgb1	Anti-inflammatory; Pro-inflammatory; DNA Damage; Autophagy; Cell Migration;	high mobility group box 1	-2.378	0.000

	Cell Adhesion; Apoptotic Cell Clearance			
Fbxo7	Autophagy; Mitophagy; Ubiquitination; Anti-Apoptotic	F-box protein 7	-1.518	0.009
Tctn3	Pro-Apoptotic; Ciliogenesis	tectonic family member 3	-1.740	0.001
Ypel3	Pro-Apoptotic; Senescence; Proliferation	yippee-like 3 (Drosophila)	-1.288	0.088
Il3ra	Proliferation; Anti-Apoptotic	interleukin 3 receptor, alpha chain	-1.743	0.007
Naa15	Angiogenesis; Anti-Apoptotic; Differentiation; Protein Acetylation	N(alpha)-acetyltransferase 15, NatA auxiliary subunit	-1.569	0.045
Rrm2b	DNA Damage; Anti-Apoptotic; Oxidative Stress	ribonucleotide reductase M2 B (TP53 inducible)	-1.469	0.078
Atf3	ER Stress/UPR; PERK; Pro-Apoptotic	activating transcription factor 3	-1.359	0.008
Dnajb1	UPR; Activates HSPA1A/B	DnaJ heat shock protein family (Hsp40) member B1	-1.624	0.081
Eps15l1	Receptor-Mediated Endocytosis; EGF Signaling	epidermal growth factor receptor pathway substrate 15-like 1	-1.464	0.063
Dnajc6	Clathrin-Mediated Endocytosis; Vesicular Transport	DnaJ heat shock protein family (Hsp40) member C6	-1.912	0.046
Slc4a10	Intracellular pH Regulation	solute carrier family 4, sodium bicarbonate cotransporter-like, member 10	-1.851	0.011
Tmem165	Lysosomal pH Regulation; N-linked Glycosylation; Calcium Ion Homeostasis	transmembrane protein 165	-1.413	0.083
Pes1	Ribosome Biogenesis; Cell Cycle	pescadillo ribosomal biogenesis factor 1	-1.370	0.010
Mtif3	Translation Regulation	mitochondrial translational initiation factor 3	-1.484	0.040
Chd6	Transcription Regulation; Chromatin Organization	chromodomain helicase DNA binding protein 6	-1.202	0.032
Gtf2a1	Transcriptional Regulation	general transcription factor II A, 1	-1.718	0.002
Phf19	Transcriptional Regulation; Proliferation/Cell Cycle	PHD finger protein 19	-2.045	0.000
Eid1	Cell Cycle; Transcription Regulation	EP300 interacting inhibitor of differentiation 1	-1.701	0.009
Ahctf1	Cell Cycle	AT hook containing transcription factor 1	-1.130	0.099
Arel1	Ubiquitination; Anti-Apoptotic	apoptosis resistant E3 ubiquitin protein ligase 1	-1.591	0.006
Fbxl15	Ubiquitination; Neddylation; Antigen Presentation	F-box and leucine-rich repeat protein 15	-1.671	0.050

Uba2	Ubiquitination; SUMOylation	ubiquitin-like modifier activating enzyme 2	-1.475	0.006
Cacna2d2	Calcium Homeostasis; Calcium Signaling	calcium channel, voltage-dependent, alpha 2/delta subunit 2	-1.463	0.083
