# Supplementary material for: Maternal diet during early gestation influences postnatal taste activity–dependent pruning by microglia
Source: J Exp Med. 2023 Sep 21;220(12):e20212476. doi: 10.1084/jem.20212476 (PMC10512853; doi:10.1084/jem.20212476)
Supplement: Table S4 — shows statistical comparisons of C1q and C3 knockout mice with controls and E3–E12 diet mice mean (±SEM). [file JEM_20212476_TableS4.pdf]

Table S4 – Statistical Comparisons of C1q and C3 Knockout Mice with Controls and E3-E12 Diet Mice -- Mean (±SEM).

IX

	P15	P30	P60
Controls	45.4(7.1)	34.9(8.4)	47.7(2.7)
E3-E12 Diet	24.2(2.5)	56.4(5.7)	43.6(11.6)
C1q KO	31.2(1.5)	43.2(5.0)	88.6(10.3)
C3 KO	44.2(11.2)	50.0(9.4)	46.1(13.6)

GSP

	P15	P30	P60
Controls	105.4(4.6)	45.1(2.9)	51.4(4.8)
E3-E12 Diet	57.3(3.0)	99.7(9.4)	106.2(4.5)
C1q KO	94.6(12.9)	94.7(8.4) p=0.001	88.5(10.3)
C3 KO	70.9(3.5) p=0.0006	73.8(9.6)	83.2(13.7)

CT

	P15	P30	P60
Controls	98.5(5.6)	63.3(6.5)	67.3(2.2)
E3-E12 Diet	65.1(4.2)	120.2(10.1)	121.3(7.8)
C1q KO	124.7(9.0) p=0.003	112.3(6.7) p=0.0005	132.6(7.8) p=0.0006
C3 KO	92.9(9.1)	88.6(6.6)	90.1(8.0)

	P15	P30	P60
Controls	n=6	n=5	n=5
E3-E12 Diet	n=6	n=5	n=5
C1q KO	n=4	n=6	n=5
C3 KO	n=5	n=5	n=5

[1]Like colored cells within each nerve X age group denotes statistical difference between controls and/or C1q and C3 knockout mice.
[2]Like colored numbers between cells for each nerve X age group denotes statistical difference between E3-E12 diet mice and/or C1q and C3 knockout mice.
