# Supplementary material for: Maternal diet during early gestation influences postnatal taste activity–dependent pruning by microglia
Source: J Exp Med. 2023 Sep 21;220(12):e20212476. doi: 10.1084/jem.20212476 (PMC10512853; doi:10.1084/jem.20212476)
Supplement: Table S5 — shows immunohistochemical statistical comparisons for C1q+ label. [file JEM_20212476_TableS5.pdf]

Table S5 – Immunohistochemical statistical comparisons for C1q+ label.

		C1q+ Within Microglia		C1q+ Outside of Microglia		Ratio Surround:Core	
	Density of Total C1q+ (X 10^3 μm^3)	Core Region	Surround Region	Core Region	Surround Region	Ratio out:in within microglia	Ratio out:in outside of microglia
**P15 Control**	139.4(16.7)	12.4(1.2)	20.1(2.5)	84.8(10.8)	137.9(17.0)	1.6(0.1)	1.6(0.1)
**P25 Control**	192.9(17.9)	9.6(1.1)	14.5(1.7)	149.7(21.4)	201.1(18.7)	1.5(0.1)	1.4(0.1)
**Adult Control**	139.4(16.7)	8.1(0.9) p=0.02	11.2(1.1) p=0.01	141.0(18.4)	186.1(26.6)	1.4(0.8)	1.3(0.0) p=0.0001
**P15 E3-E12 Diet**	199.7(16.1)	11.5(0.9)	17.1(0.7)	137.6(13.7) p=0.01	206.1(15.5) p=0.01	1.5(0.1)	1.5(0.1)
**P25 E3-E12 Diet**	216.7(12.2)	9.2(0.6)	14.5(1.3)	165.7(12.5)	220.9(14.1)	1.6(0.2)	1.3(0.3)
**Adult E3-E12 Diet**	199.7(16.1)	8.4(0.9)	12.6(1.1) p=0.008	127.9(12.5)	175.4(15.1)	1.5(0.1)	1.4(0.0)

[1]Density (Means ±SEM) of C1q+ label (X 10^3 μm^3) shown as total amount, the amount contained within microglia in the reg of the NST with the most dense label (core) and in the surround, and the amount contained outside of microglia in the region of the NST with the most dense label (core) and in the surround. The last two columns show the ratio of C1q+ label contained in microglia outside of the core to within the core and the ratio of label not contained in microglia outside of the core to within the core.

[2]Like colored numbers between cells denotes statistical difference with the p values noted on the lower mean.
